# Phylogenetic relationships of the New World titi monkeys (*Callicebus*): first appraisal of taxonomy based on molecular evidence

**DOI:** 10.1186/s12983-016-0142-4

**Published:** 2016-03-01

**Authors:** Hazel Byrne, Anthony B. Rylands, Jeferson C. Carneiro, Jessica W. Lynch Alfaro, Fabricio Bertuol, Maria N. F. da Silva, Mariluce Messias, Colin P. Groves, Russell A. Mittermeier, Izeni Farias, Tomas Hrbek, Horacio Schneider, Iracilda Sampaio, Jean P. Boubli

**Affiliations:** School of Environment and Life Sciences, University of Salford, Room 315, Peel Building, Salford, UK; Conservation International, Arlington, VA USA; Universidade Federal do Pará, Campus Universitário de Bragança, Bragança, Pará Brazil; Department of Anthropology, Institute for Society and Genetics, University of California, Los Angeles, CA USA; Evolution and Animal Genetics Laboratory, Universidade Federal do Amazonas, Manaus, Amazonas Brazil; Coleções Zoológicas, Instituto Nacional de Pesquisas da Amazônia, Manaus, Amazonas Brazil; Universidade Federal de Rondonia, Porto Velho, Rondonia Brazil; School of Archaeology & Anthropology, Australian National University, Canberra, Australia

**Keywords:** Callicebinae, Titi monkey, Genus-level taxonomy, Molecular phylogenetics, Platyrrhini, *Callicebus*, *Cheracebus*, *Plecturocebus*, Amazon, Atlantic forest

## Abstract

**Background:**

Titi monkeys, *Callicebus*, comprise the most species-rich primate genus—34 species are currently recognised, five of them described since 2005. The lack of molecular data for titi monkeys has meant that little is known of their phylogenetic relationships and divergence times. To clarify their evolutionary history, we assembled a large molecular dataset by sequencing 20 nuclear and two mitochondrial loci for 15 species, including representatives from all recognised species groups. Phylogenetic relationships were inferred using concatenated maximum likelihood and Bayesian analyses, allowing us to evaluate the current taxonomic hypothesis for the genus.

**Results:**

Our results show four distinct *Callicebus* clades, for the most part concordant with the currently recognised morphological species-groups—the *torquatus* group, the *personatus* group, the *donacophilus* group, and the *moloch* group. The *cupreus* and *moloch* groups are not monophyletic, and all species of the formerly recognized *cupreus* group are reassigned to the *moloch* group. Two of the major divergence events are dated to the Miocene. The *torquatus* group, the oldest radiation, diverged *c.* 11 Ma; and the Atlantic forest *personatus* group split from the ancestor of all *donacophilus* and *moloch* species at 9–8 Ma. There is little molecular evidence for the separation of *Callicebus caligatus* and *C. dubius,* and we suggest that *C. dubius* should be considered a junior synonym of a polymorphic *C. caligatus*.

**Conclusions:**

Considering molecular, morphological and biogeographic evidence, we propose a new genus level taxonomy for titi monkeys: *Cheracebus* n. gen. in the Orinoco, Negro and upper Amazon basins (*torquatus* group), *Callicebus* Thomas, 1903, in the Atlantic Forest (*personatus* group), and *Plecturocebus* n. gen. in the Amazon basin and Chaco region (*donacophilus* and *moloch* groups).

**Electronic supplementary material:**

The online version of this article (doi:10.1186/s12983-016-0142-4) contains supplementary material, which is available to authorized users.

## Background

Titi monkeys, *Callicebus* Thomas, 1903, are small to medium-sized (1–2 kg), New World primates of the family Pitheciidae. They comprise an old platyrrhine radiation that diverged from their sister clade, the Pitheciinae, in the Miocene, *c*. 20 Ma [1–3]. *Callicebus* species occur only in South America, with an extensive range from the foothills of the northern Andes in Colombia to the tropical forests of the Amazon and upper Orinoco basins, the Atlantic forest region of Brazil, forest patches in the xerophytic Caatinga of northeast Brazil, the wooded savanna (Cerrado) of central Brazil, and the Beni Plain in northern Bolivia, extending south as far as the Chacoan forests south and east of Santa Cruz in Bolivia and into northeast Paraguay [4–9].

*Callicebus* is the most species rich of any primate genus; 31 were listed by Ferrari et al. [10]. Two new species have been described since then, *Callicebus miltoni* Dalponte et al., 2014 and *C. urubambensis* Vermeer & Tello-Alvarado, 2015. Vermeer & Tello-Alvarado [11] also reinstated *C. toppini* Thomas, 1914, for long incorrectly considered a synonym of *C. cupreus*. These 34 titi species form a highly diverse group of primates, showing interspecific differences in body size, pelage colour, cranial dimensions, and chromosome number [5, 6, 12–14]. Kobayashi [13] employed cranial morphometrics to propose the current species-group arrangement for *Callicebus* taxa, which he suggested was consistent with variation in other characters, such as pelage colouration, karyotype, and geographic range. Species-level classification, however, has focused particularly on pelage colouration (e.g., [5, 6, 12, 15–17]), but there are evident limitations to this phenotypic system in light of the considerable intraspecific and within-population variation (e.g., [14, 18–20]). To comprehend the real taxonomic diversity of the titis, congruency is required between phenotypic traits and additional characters, such as DNA sequence data.

Some recent phylogenetic studies based on large molecular datasets have clarified high-level (genus and family) taxonomic relationships for primates [1, 2, 21]. These higher-level phylogenies reveal surprisingly deep divergence dates (Miocene) for the major *Callicebus* clades. However, most specimens were of captive origin and rather few titi species were included in these studies, limiting their usefulness in inferring species-level relationships. To date, there has been no explicit molecular investigation of the phylogenetic relationships of the *Callicebus* species and, consequently, the evolutionary history of titi monkeys remains poorly studied. The current taxonomy has yet to be tested using molecular evidence.

Here, we present a molecular phylogeny of the genus *Callicebus* based on DNA sequence data from 20 independent nuclear loci and two mitochondrial loci. In taking a molecular approach, we investigate phylogenetic relationships and divergence times among 15 species (with representatives of all species groups *sensu* Kobayashi, 1995) using concatenated Bayesian and maximum likelihood (ML) analyses. In contrast to high-level primate phylogenies (e.g., [1, 2, 21]), most of the *Callicebus* species included in this study are represented by multiple wild-caught specimens of known provenance and taxonomic identification. Taking into account the results from our phylogenetic analyses, as well as morphological and biogeographic evidence, we suggest a revised taxonomy that recognises three genera of titi monkey in the subfamily Callicebinae that are largely coherent with Kobayashi’s [13] morphological species groups. Below, we review changes to the taxonomy of the titis since Hershkovitz’s reviews [5, 12, 15].

### *Callicebus* taxonomy

*Simia* Linnaeus, C. 1758. *Syst. Nat*. 10th ed., 1: 25. In part. Humboldt, A. von. 1811. *Rec. Obs. Zool. Anat. Comp*. 1: 319. *Simia lugens* (= *Callicebus lugens*).

*Cebus* Erxleben, C. P. 1777. *Systema Regni Anim. Mammalia*, p. 44. In part. Hoffmannsegg, G. von. 1807. *Mag. Ges. Naturf. Freunde*, Berlin, 9: 97. *Cebus moloch* (= *Callicebus moloch*).

*Callitrix* Hoffmannsegg, G. von. 1807. *Mag. Ges. Naturf. Fr*., Berlin, 10: 86. Type species by monotypy *Callitrix torquata* Hoffmannsegg. Name pre-occupied by *Callitrix* Desmarest, 1804, a junior synonym of *Cebus* Erxleben, 1777.

*Callithrix* Geoffroy Saint-Hilaire, É. 1812. Suite en Tableau des Quadrumanes. *Ann. Mus. Hist. Nat. Paris*, 19: 112. Included *Callithrix sciureus* (Linnaeus) (= *Saimiri sciureus*), *Callithrix personnatus* [sic] É. Geoffroy Saint-Hilaire (= *Callicebus personatus*), *Callithrix lugens* (Humboldt), *Callithrix amictus* É. Geoffroy Saint-Hilaire, *Callithrix torquatus* (Hoffmannsegg), and *Callithrix moloch* (Hoffmannsegg). Name pre-occupied by *Callithrix* Erxleben, 1777, for the marmosets, Callitrichidae Thomas, 1903.

*Saguinus* Lesson, R. P. 1827. *Manuel de mammalogie*. J. B. Baillière, Paris: 56. Included all species listed by É. Geoffroy Saint-Hilaire (1812) for *Callithrix*, along with *Saguinus melanochir* (Weid-Neuwied) (= *Callicebus melanochir*), and *Saguinus infulatus* Kuhl (= *Aotus infulatus*). Name pre-occupied by *Saguinus* Hoffmannsegg, 1807, for the tamarins, Callitrichidae.

*Callicebus* Thomas, O. 1903. *Ann. Mag. Nat. Hist*., 7^th^ series, 12: 456. Type species *Simia personata* É. Geoffroy Saint-Hilaire, 1812.

In the 1800s, titis were generally included in the genus *Callithrix* É. Geoffroy Saint-Hilaire, 1812. Thomas [22] pointed out that the name was pre-occupied by *Callithrix* Erxleben, 1777 (the currently accepted generic epithet for the marmosets) and proposed the name *Callicebus* Thomas, 1903, which has been in use ever since.

Goodman et al*.* [23] suggested that members of the *torquatus* species group should be placed in a subgenus due to the last common ancestor with *Callicebus moloch* having an estimated age of more than 6 Ma. They suggested the name *Torquatus*. Groves [16, 17] listed *Torquatus* as a subgenus of *Callicebus* Thomas, 1903, with *Callicebus torquatus* (Hoffmannsegg, 1807), as the type species. As pointed out by Groves himself (in litt.), Goodman et al.’s [23] suggestion of the name *Torquatus*, as proposed, does not conform to the requirements of Article 13 of the *International Code of Zoological Nomenclature* (ICZN, 1999): Names published after 1930. 13.1. “To be available, every new name published after 1930 must satisfy the provisions of Article 11 and must – 13.1.1 be accompanied by a description or definition that states in words characters that are purported to differentiate the taxon, or – 13.1.2 be accompanied by a bibliographic reference to such a public statement […], or – 13.1.3 be proposed expressly as a new replacement name (*nomen novum*) for an available name […]”. Thus the name *Torquatus* is a *nomen nudum*, and unavailable.

### Species and species groups

Elliot [24], Cabrera [25], and Hill [26] listed 22–34 titi monkeys, of which 22 are considered valid taxa today. Hershkovitz [5, 12, 15] subsequently established the basis for the present classification for the genus. In 1963 [15], he recognised just 10 taxa across two polytypic species (*Callicebus moloch* and *C. torquatus*). Although the Atlantic forest *C. personatus* taxa were not included in this early review, Hershkovitz [15] suggested that they were subspecies of *C. moloch*. This view of titi monkey diversity prevailed until Hershkovitz’s revisions in 1988 and 1990. His analysis of around 1,200 museum specimens resulted in the recognition of 25 taxa across five polytypic and eight monotypic species, which he arranged in four clusters that he labelled the *modestus*, *donacophilus*, *moloch* and *torquatus* species groups (Table 1) [5, 12].

**Table 1:** The taxonomy of the titis

Hershkovitz [15]	Hershkovtiz [5, 12]	Kobayashi [13]; Kobayashi & Langguth [28]	Van Roosmalen et al. [6]	Groves [17]	Present study
**Genus ** ***Callicebus***	**Genus ** ***Callicebus***	**Genus ** ***Callicebus***	**Genus ** ***Callicebus***	**Genus ** ***Callicebus***	**Genus ** ***Cheracebus***
--	--	--	--	**Subgenus ** ***Torquatus***	--
--	***torquatus *** **group**	***torquatus *** **group**	***torquatus *** **group**	***torquatus *** **group**	--
*C. torquatus torquatus*	*C. torquatus torquatus*	*C. torquatus torquatus*	*C. torquatus*	*C. torquatus*	*C. torquatus*
*C. t. lugens*	*C. t. lugens*	*C. t. lugens*	*C. lugens*	*C. lugens*	C. lugens*
--	*C. t. lucifer*	*C. t. lucifer*	*C. lucifer*	*C. lucifer*	*C. lucifer*
--	*C. t. purinus*	*C. t. purinus*	*C. purinus*	*C. purinus*	C. purinus*
--	*C. t. regulus*	*C. t. regulus*	*C. regulus*	*C. regulus*	*C. regulus*
*C. t. medemi*	*C. t. medemi*	*C. t. medemi*	*C. medemi*	*C. medemi*	*C. medemi*
--	--	--	--	**Subgenus ** ***Callicebus***	**Genus ** ***Callicebus***
--	***moloch *** **group**	***personatus *** **group**	***personatus *** **group**	***personatus *** **group**	--
--	*C. personatus personatus*	*C. personatus*	*C. personatus*	*C. personatus*	C. personatus*
--	*C. p. melanochir*	*C. melanochir*	*C. melanochir*	*C. melanochir*	*C. melanochir*
--	*C. p. nigrifrons*	*C. nigrifrons*	*C. nigrifrons*	*C. nigrifrons*	C. nigrifrons*
--	*C. p. barbarabrownae*	*C. barbarabrownae*	*C. barbarabrownae*	*C. barbarabrownae*	*C. barbarabrownae*
--	--	*C. coimbrai*	*C. coimbrai*	*C. coimbrai*	C. coimbrai*
--	--	--	--	--	**Genus ** ***Plecturocebus***
--	--	***moloch *** **group**	***moloch *** **group**	***moloch *** **group**	***moloch *** **group**
*C. moloch moloch*	*C. moloch*	*C. moloch*	*C. moloch*	*C. moloch*	P. moloch*
--	*C. cinerascens*	*C. cinerascens*	*C. cinerascens*	*C. cinerascens*	P. cinerascens*
*C. m. hoffmannsi*	*C. hoffmannsi hoffmannsi*	*C. hoffmannsi hoffmannsi*	*C. hoffmannsi*	*C. hoffmannsi*	P. hoffmannsi*
--	*C. h. baptista*	*C. h. baptista*	*C. baptista*	*C. baptista*	*P. baptista*
--	--	--	*C. bernhardi*	*C. bernhardi*	P. bernhardi*
*C. m. brunneus*	*C. brunneus*	*C. brunneus*	*C. brunneus*	*C. brunneus*	P. brunneus*
--	*--*	***cupreus *** **group**	***cupreus *** **group**	--	--
*C. m. cupreus*	*C. cupreus cupreus*	*C. cupreus cupreus*	*C. cupreus*	*C. cupreus*	P. cupreus*
*C. m. discolor*	*C. c. discolor*	*C. c. discolor*	*C. discolor*	*C. discolor*	*P. discolor*
*C. m. ornatus*	*C. c. ornatus*	*C. c. ornatus*	*C. ornatus*	*C. ornatus*	*P. ornatus*
--	*C. caligatus*	*C. caligatus*	*C. caligatus*	*C. caligatus*	P. caligatus*
--	*C. dubius*	*C. dubius* ^a^	*C. dubius*	*C. dubius*	--
--	--	--	*C. stephennashi*	*C. stephennashi*	*P. stephennashi*
--	--	--	--	--	*P. aureipalatii*
--	--	--	--	--	*P. caquetensis*
--	--	--	--	--	*P. vieirai*
--	--	--	--	--	P. miltoni*
--	--	--	--	--	*P. toppini*
--	***donacophilus *** **group**	***donacophilus *** **group**	***donacophilus *** **group**	***donacophilus*** **group**	***donacophilus *** **group**
*C. m. donacophilus*	*C. donacophilus donacophilus*	*C. donacophilus donacophilus*	*C. donacophilus*	*C. donacophilus*	P. donacophilus*
--	*C. d. pallescens*	*C. d. pallescens*	*C. pallescens*	*C. pallescens*	*P. pallescens*
--	*C. oenanthe*	*--*	*C. oenanthe*	*C. oenanthe*	*P. oenanthe*
--	*C. olallae*	*C. olallae*	*C. olallae*	*C. olallae*	*P. olallae*
--	--	--	--	--	*P. urubambensis*
--	***modestus *** **group**	--	--	***modestus *** **group**	--
--	*C. modestus*	*C. modestus*	*C. modestus*	*C. modestus*	*P. modestus*
10 taxa	25 taxa	25 taxa	28 taxa	28 taxa	33 taxa

Taxonomic arrangement for *Callicebus* taxa as proposed by Hershkovitz [15]; Hershkovitz [5, 12]; Kobayashi [13] and Kobayashi & Langguth [28]; Van Roosmalen *et al*. [6]; Groves [17]; and the present study. Classification for species not included in this study follows Groves [17], and species described and reinstated after Groves [11, 17, 66, 94–96] with the exception of *P. modestus* where we follow Kobayashi [13]. Bold indicates species' classification

^a^Species group undetermined

*Species included in this study

To infer phylogenetic relationships, Kobayashi [13] carried out a morphometric analysis of cranial measurements for 23 taxa, and modified Hershkovitz’s [5, 12] species groups. He maintained the *torquatus* and *donacophilus* groups, but included *C. modestus* in the latter. He split the *moloch* group into three: the *personatus* group, the *moloch* group and the *cupreus* group (Table 1). As other characters, such as pelage colouration, karyotype, and geographic range, were consistent with this classification, he argued that these groups represented phylogenetically independent clades. Kobayashi [13] suggested that the *donacophilus*, *moloch*, and *cupreus* groups were closely related, while the *personatus* and *torquatus* groups presented a higher degree of character differentiation. Based upon the occlusal pattern of the upper molars, the *torquatus* group was proposed as the earliest lineage [27].

The distinctiveness of the *torquatus* group has long been recognised; *C. torquatus* was one of the two species in Hershkovitz’s first appraisal in 1963 [15]. He considered it polytypic, with three subspecies: *C. t. torquatus* (Hoffmannsegg, 1807); *C. t. lugens* (Humboldt, 1811); and *C. t. medemi* Hershkovitz, 1963. Hershkovitz [5, 12] subsequently resurrected three other taxa: *lucifer* Thomas, 1914; *regulus* Thomas, 1927; and *purinus* Thomas, 1927—all as subspecies of *torquatus*. As of 1990, therefore the *torquatus* group consisted of a single species with six subspecies. Groves [16] listed *medemi* as a species, but otherwise followed Hershkovitz in maintaining the remaining forms as subspecies of *torquatus*. Van Roosmalen et al. [6] and Groves [17] classified all members of the *torquatus* group as species. Taking note of the suggestion of Goodman et al*.* [23], Groves [17] placed the members of the *torquatus* group in the subgenus *Torquatus* (all other titis in the subgenus *Callicebus*), although, as mentioned, he subsequently realised that the name as suggested by Goodman et al. [23] was a *nomen nudum*.

Hershkovitz [12] recognised three subspecies of *C. personatus; C. p. personatus* (É. Geoffroy Saint-Hilaire, 1812); *C. p. melanochir* (Wied-Neuwied, 1820); and *C. p. nigrifrons* (Spix, 1823). He indicated that they could be considered subspecies of *C. moloch*, and placed them in his *moloch* species group [5, 12]. In his 1990 revision, he described another subspecies from northeast Brazil, *C. p. barbarabrownae* [5]. Kobayashi [13] continued to recognise these four titis as subspecies but placed them in a separate species group, based on the high degree of character differentiation between *C. personatus* and other *Callicebus* taxa. Kobayashi & Langguth [28] described *C. coimbrai*, a member of the *personatus* group from northeast Brazil, and determined that all members of the *personatus* group be considered distinct species.

The craniometric study of Kobayashi [13] showed that the *donacophilus*, *moloch*, and *cupreus* groups are more closely related to each other than they are to the *torquatus* and *personatus* groups. This is reflected in the early history of their taxonomy. Hershkovitz [15] recognised a single species with seven subspecies in his *moloch* group: *C. moloch moloch* (Hoffmannsegg, 1807); *C. m. cupreus* (Spix, 1823); *C. m. donacophilus* (d’Orbigny, 1836); *C. m. brunneus* (Wagner, 1842); *C. m. discolor* (I. Geoffroy & Deville, 1848); *C. m. ornatus* (Gray, 1866); and *C. m. hoffmannsi* Thomas, 1908. Hershkovitz’s subsequent revisions [5, 12] resulted in the description of a new species, *dubius* Hershkovitz, 1988, and the reinstatement of *cinerascens* Spix, 1823, *caligatus* Wagner, 1842, *modestus* Lönnberg, 1939, *olallae* Lönnberg, 1939, *baptista* Lönnberg, 1939, *pallescens* Thomas, 1907, and *oenanthe* Thomas, 1924, as valid taxa. Excluding the *C. personatus* subspecies, Hershkovitz [5] listed 15 species and subspecies, and classified them into three species groups; the *modestus* group, the *donacophilus* group, and the *moloch* group (Table 1). Groves [16] maintained the species groups of Hershkovitz [5], but raised all the *donacophilus* and *moloch* (but not *C. personatus*) group members to species. In his review, Groves questioned the distinction between *C. cupreus*, *C. caligatus*, *C. discolor*, and *C. dubius*, and placed the latter three as synonyms of *C. cupreus*. Groves, however, subsequently accepted them as valid species [17].

The current taxonomic arrangement was established in the review by Van Roosmalen et al*.* [6]. They followed the species groups proposed by Kobayashi [13] but listed all recognised taxa as species, as proposed by Groves [16] (see also [28]). Van Roosmalen et al. [6] described *C. bernhardi* and *C. stephennashi*, belonging to the *moloch* and *cupreus* groups, respectively. Five new species have been described since 2002; *C. aureipalatii* Wallace et al., 2006, *C. caquetensis* Defler et al., 2010, *C. vieirai* Gualda-Barros et al., 2012, *C. miltoni* Dalponte et al., 2014, in the *moloch* and *cupreus* groups, and *C. urubambensis* Vermeer & Tello-Alvarado, 2015, assigned to the *donacophilus* group. Vermeer & Tello-Alvarado [11] also reinstated *C. toppini* Thomas, 1914, as a member of the *cupreus* group.

## Results

### Group-level topology

**Fig. 1:**
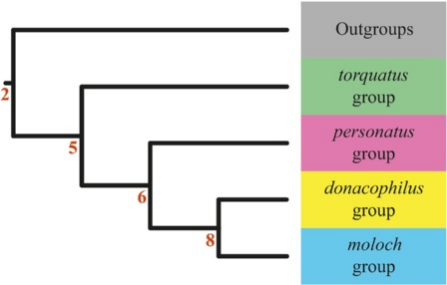
Phylogenetic reconstruction showing *Callicebus* species-group level topology found across all datasets. All nodes were significantly supported in all analyses (BP ≥ 70 % and PP ≥ 0.95). Node numbers correspond to those in Fig. 2, 3 and are listed with support values for all analyses in Additional file 1

All analyses across the mitochondrial, nuclear and combined datasets yielded an identical topology for the *Callicebus* species groups (Fig. 1). Our results support the division of *Callicebus* into four reciprocally monophyletic groups; the *torquatus* clade, here including *C. lugens* and *C. purinus*; the *personatus* clade with *C. personatus*, *C. coimbrai*, and *C. nigrifrons*; the *donacophilus* clade with *C. donacophilus*; and the *moloch* clade containing all remaining taxa (*C. hoffmannsi*, *C. cinerascens*, *C. miltoni*, *C. bernhardi*, *C. moloch*, *C.* cf. *moloch*, *C. brunneus*, *C. cupreus*, *C. dubius*, and *C. caligatus*). The *torquatus* group is strongly supported as the earliest radiation to diverge. It is followed by the separation of the *personatus* group from the *donacophilus*-*moloch* clade, with the final group-level split occurring between the *donacophilus* group and the *moloch* group. These major diversification events receive significant support across all analyses (bootstrap percentage, BP > 70 %; posterior probability, PP > 0.95), and thus, our results suggest a highly resolved topology for the *Callicebus* species groups (Fig. 1). As Kobayashi’s *moloch* and *cupreus* groups were not monophyletic, we adopt Groves’ [17] classification and include all *cupreus* group species (*sensu* Kobayashi, 1995) in the *moloch* group. A summary of node support per analysis is presented in Additional file 1.

### Species-level topology

Within each dataset, ML (RAxML) and Bayesian (MrBayes, BEAST) inference trees all presented similar species-level topologies. Individual trees with node support values for each analysis are found in Additional files 2 (combined dataset), 3 (nuclear dataset) and 4 (mitochondrial dataset).

**Fig. 2:**
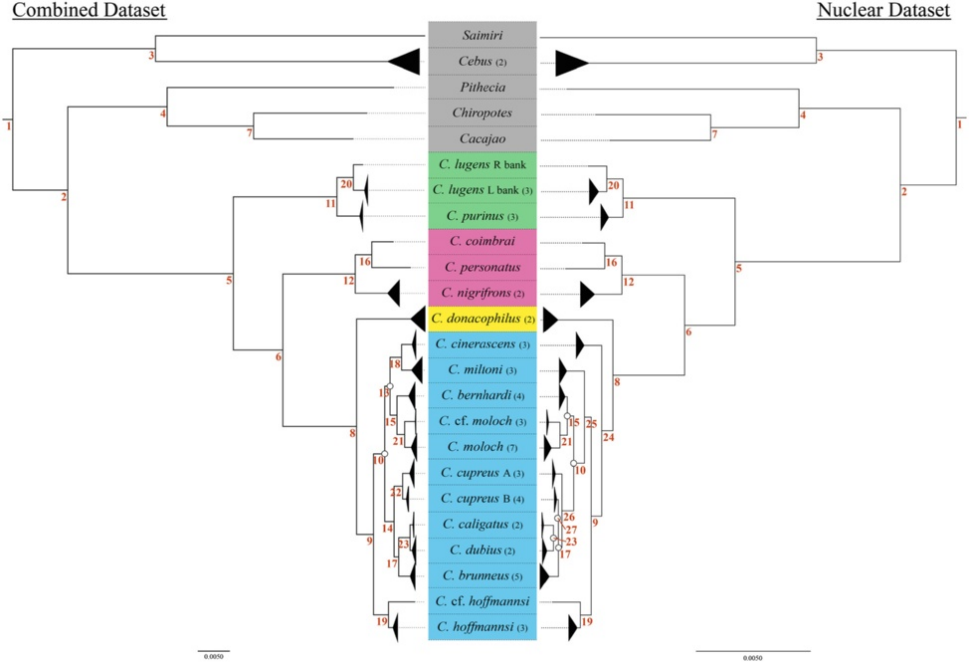
Molecular phylogeny showing relationships among *Callicebus* taxa based on 53 *Callicebus* and 6 outgroup individuals. Shown are maximum likelihood trees inferred from the combined dataset (left) and the nuclear dataset (right), with branches collapsed to represent clades of interest. Numbers in parenthesis indicate the number of individuals represented in the collapsed clade. See Additional file 2 and 3 for the expanded ML (RAxML) and Bayesian (MrBayes, BEAST) trees with node support values. Unmarked nodes were significantly supported in all analyses (BP ≥ 70 % and PP ≥ 0.95), while nodes marked with white circles received low support (BP < 70 % and/or PP < 0.95). Red numbers represent nodes of interest listed with support values for all methods of analysis in Additional file 1. Background colours reflect species group; green for the *torquatus* group, pink for the *personatus* group, yellow for *donacophilus* group, blue for the *moloch* group; and grey indicates the outgroup species

The phylogenetic relationships among taxa in the *torquatus* and *personatus* clades are identical for all three datasets (Fig. 2). All nodes have significant support (BP > 70 %, PP > 0.95) with the exception of the sister-relationship between *C. lugens* from the left and right banks of the Rio Negro, which is not supported for the mitochondrial dataset (BP = 64 %, PP = 0.78). *Callicebus donacophilus* is consistently supported as an independent radiation, sister to the *moloch* species group. Species-level relationships within the *moloch* group, however, vary according to each dataset. The principal differences were found between the combined and nuclear dataset topologies in the phylogenetic position of *C. cinerascens* and *C. miltoni*, as well as the phylogenetic relationships of *C. cupreus* and other closely related species (Fig. 2). The mitochondrial trees largely reflect those inferred from the combined dataset except in the phylogenetic position of *C. hoffmannsi* (see Additional file 4), which is discussed below.

### The *moloch* group

In contrast to morphological hypotheses [6, 13], the *moloch* and *cupreus* groups were not monophyletic; *C. hoffmannsi* does not share a most recent common ancestor with other species of *moloch* group (*sensu* Kobayashi, 1995); and *C. brunneus* of the *moloch* group (*sensu* Kobayashi, 1995) is nested in the *cupreus* species group clade.

There is little molecular evidence for the separation of specimens identified as *C. caligatus* and *C. dubius*. The mitochondrial dataset supports *C. caligatus* and *C. dubius* as a monophyletic group (BP = 91 %, PP = 1.00); however, the two *C. dubius* do not form a clade, and branch off independently at the base of the *C. caligatus* clade. Most of the nodes within this clade are not well supported (BP < 70 %, PP < 0.95), and the topology suggests that these taxa form one, not two, species. For the nuclear and combined datasets, *C. dubius* is monophyletic and is a minimally diverged sister taxon of *C. caligatus*. A divergence matrix based on the 1140 bp cytochrome *b* gene (Additional file 5) shows genetic distance values of 0.01–0.06 between the six *C. dubius* and *C. caligatus* specimens. These values are comparable to the divergence between specimens of *C. brunneus* (0.0–0.08) or of *C. cupreus* (0.02–0.19), rather than the genetic distances found between *C. brunneus*, *C. cupreus* and the *C. caligatus*/*C. dubius* complex (0.24–0.38).

All datasets support a west-Amazonian species complex that comprises *C. brunneus, C. cupreus, C. caligatus* and *C. dubius*, and is subdivided into four distinct clades: *C. brunneus*; *C. cupreus* A; *C. cupreus* B; and *C. caligatus/C. dubius*. The sister-group relationship of *C. cupreus* (*C. cupreus* A, *C. cupreus* B) to the group comprising *C. brunneus* and *C. caligatus/dubius* is consistently supported in the combined/mitochondrial phylogeny (BP > 84 %, PP = 1.00). In the nuclear dataset, *C. cupreus* is paraphyletic and *C. cupreus* A is supported as the first diverging member of the group (BP = 95 %, PP = 1.00). The RAxML and BEAST topologies show that *C. brunneus* is the next taxon to diverge (BP = 58 %, PP = 1.00), with *C. cupreus* B being sister to *C. caligatus/dubius* (BP = 23 %, PP = 0.38). However, the MrBayes tree inferred from the nuclear dataset shows a polytomy among *C. brunneus, C. caligatus/dubius* and *C. cupreus* B*.*

*Callicebus hoffmannsi* is strongly supported as an early diverging lineage in the nuclear (between the *C. cinerascens* and *C. miltoni* radiations) and combined (as sister-group to all other species of the *moloch* group) dataset analyses. The phylogenetic relationship of *C. hoffmannsi* differs in the mitochondrial dataset (see Additional file 4), but has no statistical support (RAxML, BP = 28 %; MrBayes, unresolved polytomy).

All analyses support a clade that contains *C. moloch*, *C.* cf. *moloch* and *C. bernhardi*, with a sister-species relationship between *C. moloch* and *C.* cf. *moloch*. All nodes within this group are significantly supported (BP > 70 %, PP > 0.95) with the exception of the split between *C. bernhardi* and *C. moloch*/*C.* cf. *moloch* for the ML nuclear phylogeny (BP = 56 %, PP > 0.99). *Callicebus cinerascens* + *C. miltoni* are a sister-group to this clade in the mitochondrial (with significant support) and combined (supported only in the BEAST analysis, PP = 1.00) datasets. In the nuclear dataset, *C. cinerascens* and *C. miltoni* find significant support as independent early radiations, along with *C. hoffmannsi*. Thus, there is a conflict in the phylogenetic signals of the nuclear and mitochondrial datasets, which is reflected by low support in combined dataset, but high support in independent mitochondrial and nuclear analyses. The phylogenetic position of *C. cinerascens* and *C. miltoni*, therefore, remains unresolved.

### Divergence-time estimates

**Fig. 3:**
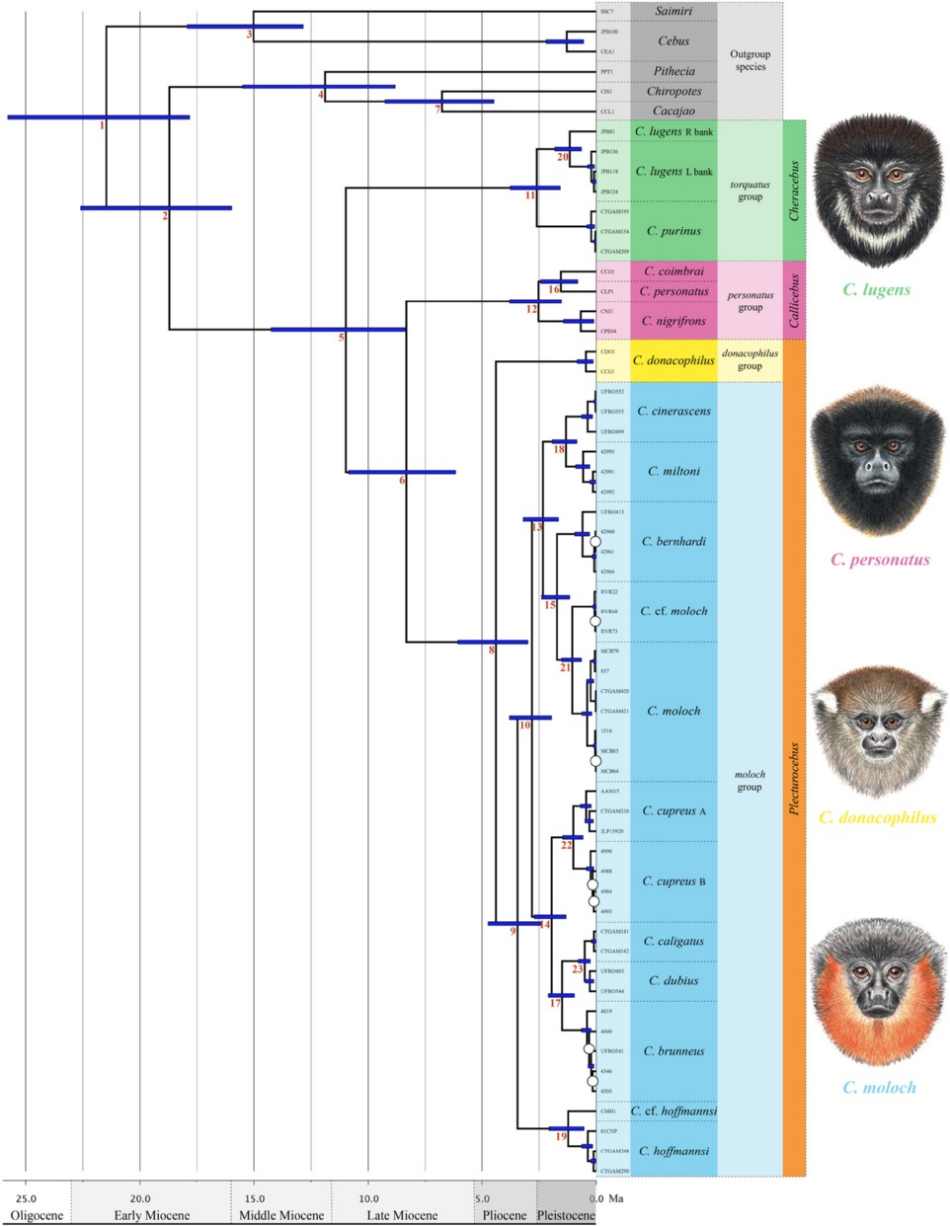
A time-calibrated phylogeny showing estimated divergence ages among *Callicebus* individuals based on the combined dataset. Unmarked nodes were strongly supported (PP ≥ 0.99), while nodes marked with white circles received low support (PP < 0.95). Node bars indicate the 95 % highest posterior density. Red numbers represent nodes of interest listed with specific support values and estimated divergence times in Additional file 1. For trees with support values and estimated divergence times for all nodes see Additional file 2 (C) and 6, respectively. Nodes numbered 2 and 3 were used for calibration. A time scale in million years and the geological periods are given. Background colours reflect species-group; green for the *torquatus* group, pink for the *personatus* group, yellow for *donacophilus* group, blue for the *moloch* group; and grey indicates the outgroup species. Illustrations by Stephen D. Nash ©Conservation International

From the combined dataset (Fig. 3, Additional file 6), we estimated the origin of crown Pitheciidae at *c.* 21.47 Ma (95 % HPD = 17.82–25.78) and the origin of crown *Callicebus* to be in the early Miocene, *c.* 18.71 Ma (95 % HPD = 15.97–22.6). The most recent common ancestor of extant *Callicebus* lineages is estimated to have lived in the late Miocene (10.98 Ma; 95 % HPD = 8.36–14.25); this ancestor gave rise to the progenitor of the *torquatus* species group (Amazon and Orinoco) and the progenitor of all other *Callicebus* clades. Next to diverge was the Atlantic forest *personatus* group at around 8.34 Ma (95 % HPD = 6.18–10.86), also in the late Miocene. The final group-level divergence is estimated to have occurred in the Pliocene, around 4.39 Ma (95 % HPD = 2.99–6.08), between *C. donacophilus* (representative of the *donacophilus* group) and the *moloch* group. In the *moloch* group, *C. hoffmannsi* diverged at an estimated 3.44 Ma (95 % HPD = 2.39–4.74), followed by the divergence of an east-Amazonian clade (*C. cinerascens, C. miltoni, C. bernhardi, C. moloch, C.* cf. *moloch*) and a west-Amazonian clade (*C. cupreus, C. brunneus, C. dubius, C. caligatus*) at around 2.81 Ma (95 % HPD = 1.95–3.8).

Sister-species divergences are estimated at 3–1 Ma for all *Callicebus* taxa included in the dating analyses. These are especially recent for species of the *moloch* group, with all sister-species splits occurring 2 − 1 Ma with the exception of the *C. dubius* and *C. caligatus* divergence, which occurred more recently at *c.* 0.5 Ma (95 % HPD = 0.26–0.79). Our dating analyses also suggest relatively deep divergences within some taxa; *C. lugens* from the left and right banks of the Rio Negro diverged around 1.16 Ma (95 % HPD = 0.65–1.82); *C. moloch* and *C.* cf. *moloch* diverged an estimated 1.05 Ma (95 % HPD = 0.64–1.52); and *C. cupreus* A and *C. cupreus* B split at around 1 Ma (95 % HPD = 0.58–1.47).

We also dated the phylogeny based on nuclear loci only (Additional file 7). Importantly, we estimated the age of divergence of *C. cinerascens* and *C. miltoni* from their sister-clades in the *moloch* group at *c.* 4.38 Ma (95 % HPD = 1.96–7.63) and 3.08 Ma (95 % HPD = 1.33–5.37), respectively. Note that *C. cinerascens* and *C. miltoni* are weakly supported as a sister-group to the *C. bernhardi*/*C. moloch*/*C.* cf. *moloch* clade in the combined dataset analyses due to mitochondrial DNA signal.

Divergence dates inferred for the combined dataset BEAST analyses are consistently slightly younger across *Callicebus* than for the dating analyses based on the nuclear loci (Table 2). A summary of node support, divergence date estimates and 95 % HPD intervals for the combined and nuclear dataset BEAST analyses is presented in Additional file 1.

**Table 2:** Estimated divergence times inferred from the combined and nuclear datasets for *Callicebus* species groups

Clade or Split	Node	Combined dataset			Nuclear dataset		
		Mean age (Ma)	Lower 95 % HPD	Upper 95 % HPD	Mean age (Ma)	Lower 95 % HPD	Upper 95 % HPD
Crown Pitheciidae	1	21.47	17.82	25.78	22.89	17.82	28.92
Pitheciinae vs. Callicebinae	2	18.71	15.97	22.6	19.13	15.93	23.8
*torquatus* group vs. *personatus* + *donacophilus* + *moloch* groups	5	10.98	8.36	14.25	12.03	7.78	16.72
*personatus* group vs. *donacophilus* + *moloch* groups	6	8.34	6.18	10.86	8.94	5.52	13.07
*donacophilus* group vs. *moloch* group	8	4.39	2.99	6.08	5.33	2.58	8.78

*Abbreviations: Ma* millions of years ago, *HPD* highest posterior density

## Discussion

In this study, we assembled one of the largest molecular datasets for any group of platyrrhine primates, sequencing 20 nuclear and two mitochondrial loci totalling over 14,000 base pairs, and including representatives of all the major callicebine lineages. Using this dataset, we provide the first comprehensive review of the Callicebinae using molecular data to assess phylogenetic relationships and divergence dates among the major lineages and to test morphological taxonomical hypotheses.

Our analyses show that *Callicebus* is divided into three principal clades of Miocene origin, corresponding to Kobayashi’s [13] *torquatus* and *personatus* groups, and a clade containing the *donacophilus*, *moloch* and *cupreus* species groups. All phylogenetic analyses yielded identical phylogenetic relationships among these three clades with estimated divergence times being in the late Miocene. Goodman et al. [23] produced time-based taxonomic classification criteria and proposed that primate species that diverged from 11 to 7 Ma be recognised as separate genera. Based on the results from our phylogenetic and divergence-time analyses, and also morphological, ecological and biogeographical (see below) evidence, we therefore suggest the division of titi monkeys into three genera in the subfamily Callicebinae (Table 1).

### A proposal for a new taxonomy of the titi monkeys at the genus level

#### Cheracebus new genus

urn:lsid:zoobank.org:act:DE67E93E-89A3-47C1-BAF3-E183F3448520

Type species. *Cheracebus lugens* (Humboldt, 1811) Widow Monkey

*Simia lugens* Humboldt, A. von. 1811. *Rec. Obs. Zool. Anat. Comp.* 1: 319.

We did not suggest the earlier named *Callitrix* [*sic*] *torquata* Hoffmannsegg, 1807, as the type species, because the original type locality given by Schlegel [29] (p. 235) is outside the range of *torquatus* as defined by Hershkovitz [5], and there is a certain, as yet unresolved, confusion concerning the diagnostic phenotypic traits for the species’ identification [18, 19, 30]. There is, as such, a lack of clarity regarding its diagnostic characteristics, its distribution, and even its validity as a taxon. Humboldt’s anecdote about *Simia lugens* was the inspiration for the name *Cheracebus* (see below).

Etymology: “*Chera*” is the Latin form of *χηρα*; Greek for “widow”. “*Cebus*” comes from the Greek “*kebos*”, which means “long-tailed monkey”. Humboldt [31, 32] referred to it as the “viudita” of the Orinoco and recounted that missionaries called it the widow monkey because of its pelage colouration—a pale face, white collar, and white hands contrasting with an overall blackish pelage—that was reminiscent of the white veil, neckerchief, and gloves of a widow in mourning. The name persevered [33] and in French it has been called the “veuve” [26, 31]. A synonym of *Simia lugens* is *Saguinus vidua* Lesson, 1840: 165. “*Vidua*” is Latin for widow.

*"The saimiri, or titi of the Orinoco, the atele, the sajou, and other quadrumanous animals long known in Europe, form a striking contrast, both in their gait and habits, with the macavahu, called by the missionaries viudita, or ‘widow in mourning’. The hair of this little animal is soft, glossy, and of a fine black. Its face is covered with a mask of a square form and a whitish colour tinged with blue. This mask contains the eyes, nose, and mouth. The ears have a rim: they are small, very pretty, and almost bare. The neck of the widow presents in front a white band, an inch broad, and forming a semicircle. The feet, or rather the hinder hands, are black like the rest of the body; but the fore paws are white without, and of a glossy black within. In these marks, or white spots, the missionaries think they recognize the veil, the neckerchief, and the gloves of a widow in mourning. The character of this little monkey, which sits up on its hinder extremities only when eating, is but little indicated in its appearance.”* [32] (p. 212).

**Fig. 4:**
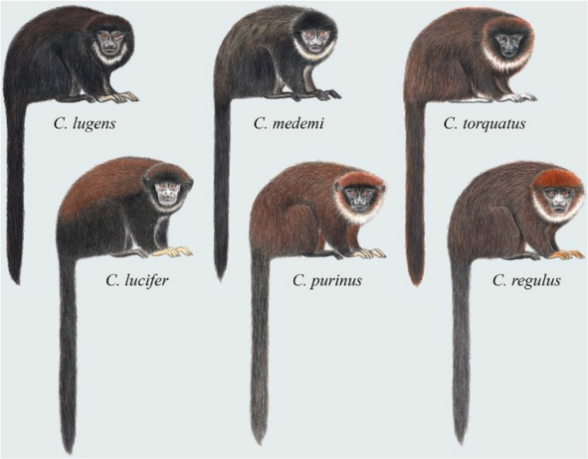
Titi monkeys, genus *Cheracebus*. Illustrations by Stephen D. Nash ©Conservation International

Distinguishing characters: *Cheracebus* comprises the *torquatus* group titis as defined by Hershkovitz [5, 12, 15], Kobayashi [13] and Groves [16] (Fig. 4). Hershkovitz’s [5] review contains detailed descriptions of the dental, cranial and post-cranial characters which distinguish the *torquatus* group, and hence, now the genus *Cheracebus*, from all other titi monkeys. He described the diagnostic characters as follows: “Average size larger than that of other species except *C. personatus* (Tables eleven, thirteen), ethmoturbinal I larger, projecting farther behind than the maxilloturbinal bone […] average cerebral index high (Table nine) [29 % of greatest skull length], diploid chromosome number = 20 (subspecies unknown) [see below], forehead, forearms, sideburns, feet, and tail blackish; crown reddish, reddish brown, mahogany, or blackish; sideburns little projecting; throat collar whitish or buffy, sometimes not well defined or absent; hands blackish, buffy, yellowish, or orange; upper parts from crown to tail base reddish brown, conspicuously to faintly banded or uniformly colored; chest, belly uniformly reddish, reddish brown, or blackish” [5] (p. 78).

Jones and Anderson [34] summarised the diagnostic characters in a taxonomic key distinguishing *Callicebus personatus* from *Callicebus torquatus* and *Callicebus moloch*, based on Hershkovitz [15]: “Color of body reddish to black, venter either not or slightly defined from dorsum; hind feet and tail to tip black; forearms black above and below; upper surface of forefeet either whitish or blackish like the wrists”.

According to Kobayashi [27], the occlusal pattern of the upper molars is relatively smooth and simple in the *torquatus* group.

Groves [16] (p. 176 − 177) added that the mesostyle and distostyle on the upper premolars are well defined, whereas in the other species-groups they are absent on P^2^ and weak or absent on P^3-4^; an entepicondylar foramen is present that is lacking in all other species; and the limbs are very long: arm 67 − 73 % of trunk length, leg 90 %. Groves [16] did not agree with Hershkovitz’s [5] assertion that the *torquatus* group titis are unusually large. The estimated time of divergence of *Cheracebus* from all other titis is 11 million years in the Middle Miocene.

**Fig. 5:**
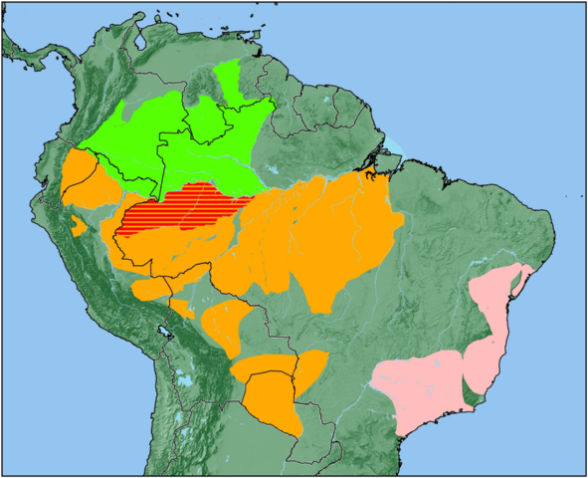
The geographic distribution of *Cheracebus* (green), *Callicebus* (pink) and *Plecturocebus* (orange). The area of sympatry between species of *Cheracebus* and *Plecturocebus* is shown in red

Geographic range: Titis of the genus *Cheracebus* occur in the Amazon and Orinoco basins, in Brazil, Colombia, Ecuador, Peru, and Venezuela (Fig. 5). North of the Solimões-Amazonas, they occur east as far as the Rio Branco in Brazil, extending into Venezuela as far north as the Rio Orinoco, west of the Río Caroni to the foothills of the Eastern Cordillera of the Andes, south of the upper Río Guaviare, Colombia, through Ecuador, north of the Río Aguarico, and into Peru to the north of the ríos Amazonas and Tigre. South of the Solimões-Amazonas, they extend eastward from the Rio Javari in Brazil, across the lower and middle rios Juruá and Purus, to the Rio Madeira [5, 17, 19, 35, 36]. In Ecuador and Peru, and Brazil south of the Rio Amazonas-Solimões, titis of this genus are sympatric with a number of the smaller titis of Hershkovitz’s [5, 12] *moloch* group.

*Cheracebus lugens* (Humboldt, 1811). Widow monkey, White-chested titi

*Simia lugens* Humboldt, A. von. 1811. *Rec. Obs. Zool. Anat. Comp.* 1: 319.

Type locality: Near San Francisco de Atabapo, at the confluence of the ríos Orinoco and Guaviare, Amazonas, Venezuela.

*Cheracebus medemi* (Hershkovitz, 1963). Black-handed titi, Medem’s titi

*Callicebus torquatus medemi.* Hershkovitz, P. 1963. *Mammalia* 27(1): 52.

Type locality: Río Meceya, near mouth, right bank Río Caquetá, Putumayo, Colombia: altitude approximately 180 m.

*Cheracebus torquatus* (Hoffmannsegg, 1807). Collared titi, white-collared titi

*Callitrix* [sic] *torquatus* Hoffmansegg, G. von. 1807. *Mag. Ges. Naturf. Fr*., Berlin 10: 86.

Type locality: Codajás, north bank Rio Solimões upstream the mouth of the Rio Negro, Amazonas, Brazil [15].

*Cheracebus lucifer* (Thomas, 1914). Yellow-handed titi

*Callicebus lucifer* Thomas, O. 1914. *Ann. Mag. Nat. Hist.*, 8^th^ ser. 13: 345.

Type locality: Yahuas, N. of Loreto, about 2°40'S, 70°30'W, Alt. 500 ft. (Thomas, 1914). Yahuas territory, near Pebas, Loreto, Peru, about 125 m [5].

*Cheracebus purinus* (Thomas, 1927). Rio Purus titi

*Callicebus purinus* Thomas, O. 1927. *Ann. Mag. Nat. Hist*. 9^th^ ser. 19: 509.

Type locality: Ayapuá, lower Rio Purus, southern affluent of Rio Solimões, Brazil.

*Cheracebus regulus* (Thomas, 1927). Juruá collared titi

*Callicebus regulus* Thomas, O. 1914. *Ann. Mag. Nat. Hist*. 9^th^ ser. 19: 510.

Type locality: Fonte Boa, upper Rio Solimões, Amazonas, Brazil.

#### Callicebus Thomas, 1903

Thomas, O. 1903. *Ann. Mag. Nat. Hist*., 7^th^ series, 12: 456. Type species. *Simia personata* É. Geoffroy Saint-Hilaire, 1812.

Type species. *Simia personata* Geoffroy Saint-Hilaire, É. 1812. In: Humboldt, 1812. *Rec. Obs. Zool.*, p. 357.

Etymology: “Calli” is from the Greek *kalos*, which means “beautiful”. “Cebus” is from the Greek *kebos*, which means “a long-tailed monkey”.

**Fig. 6:**
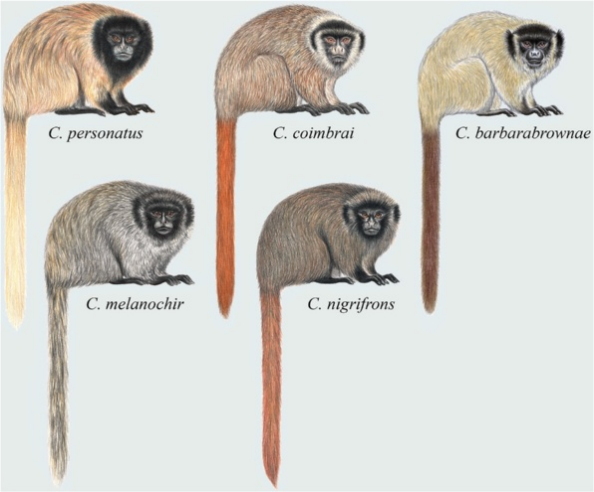
Titi monkeys, genus *Callicebus*. Illustrations by Stephen D. Nash ©Conservation International

Distinguishing characters: The genus *Callicebus* is here restricted to the Atlantic forest titis that were listed as subspecies of *C. personatus* in the *moloch* group by Hershkovitz [5], and as members of a distinct *C. personatus* group by Kobayashi [13] and Groves [16] (Fig. 6). Groves [16] included *C. coimbrai* Kobayashi & Langguth, 1999. Hershkovitz’s [5] review contains detailed descriptions of the dental, cranial and post-cranial skeletal characters which distinguish *C. personatus* from all other titi monkeys (see also [13] for craniometric differences). Hershkovitz [5] (p. 70 − 71) diagnosed *C. personatus* as follows: “Average size largest […]; cranial characters essentially as in *moloch* group except average cerebral index greater, average brain case index less […]; pelage coarse, shaggy with full coat of hidden brownish wool hairs; color of trunk variable, cover hairs with 2 or 4 pheomelanic bands sharply defined to shadowy, or uniformly, pheomelanin; cheiridia blackish, the blackish often extending proximally as a tapered band to mid-arm or mid-foreleg, remainder of limbs grayish, buffy, yellowish or orange, the hairs banded or unbanded; facial hairs long, often comparatively thick but not concealing skin; forehead blackish with or without fine buffy banding; sideburns and ear tufts blackish; tail orange, reddish, mahogany, or mixed with blackish, never entirely blackish.”

Jones & Anderson [34] summarised the diagnostic characters in a taxonomic key distinguishing *Callicebus personatus* from *Callicebus torquatus* and *Callicebus moloch*, based on Hershkovitz [15]: “Distal portion of limbs (at least forefeet and hind feet) black and in sharp contrast to the gray or rufous of wrists and other proximal parts”.

According to Kobayashi [27], the *personatus* group shows the most uneven and variable occlusal pattern in the upper molars, with the largest number of small cusps and conules.

*Callicebus coimbrai*, not included by Hershkovitz [5], conforms. It has a black forehead, crown, and ears, and a buffy body; pale cheek whiskers, the colour extending to the nape; hands and feet blackish, tail orange, and zebra stripes on the upper back [16]. The diagnostic features of the *personatus* group given by Groves [16] (p. 175) summarised Hershkovitz [5]. Estimated time of divergence *c.* 8.3 million years, in the Late Miocene.

Geographic range: Endemic to Brazil (Fig. 5). These titis are known from northeastern Brazil, south of the Rio São Francisco in forest patches in the Caatinga (*barbarabrownae*) and Atlantic forest (*coimbrai*), south through the Atlantic forest of the states of Bahia, Espírito Santo, and Rio Janeiro, west as far as the rios Paraná and Paranaíba, and south to the Rio Tieté in the state of São Paulo [5, 6, 37].

*Callicebus personatus* (É. Geoffroy Saint-Hilaire, 1812). Masked titi

*Simia personata* Geoffroy-Saint Hilaire, É. 1812. In: Humboldt, 1812. *Rec. Obs. Zool.*, p. 357.

Type locality: Brazil. Restricted by Hershkovitz [5] to the lower Rio Doce, Espírito Santo, Brazil.

*Callicebus coimbrai* Kobayashi & Langguth, 1999. Coimbra-Filho’s titi

*Callicebus coimbrai* Kobayashi, S. & Langguth, A. 1999. *Revta. Bras. Zool*. 16(2): 534.

Type locality: Proximity of the small village of Aragão, in the region of Santana dos Frades about 11.0 km SW of Pacatuba, south of the estuary of the Rio São Francisco, state of Sergipe, Brazil. 10°32'S, 36°41'W, altitude 90 m.

*Callicebus barbarabrownae* Hershkovitz, 1990. Blond titi

*Callicebus personatus barbarabrownae* Hershkovitz, P. 1990. *Fieldiana, Zool., n.s.*, (55): 77.

Type locality: Lamarão, Bahia, Brazil, altitude about 300 m above sea level.

*Callicebus melanochir* (Wied-Neuwied, 1820). Southern Bahian titi

*Callithrix melanochir* Wied-Neuwied, M. A. P. von. 1820. *Reise nach Brasilien in den Jahren 1815 bis 1817.* Vol. 1. H. L. Bronner, Frankfurt am Main, p. 258 and fn.

Type locality: Morro d’Árara or Fazenda Arara, state of Bahia, Brazil [5].

*Callicebus nigrifrons* (Spix, 1823). Black-fronted titi

*Callithrix nigrifrons* Spix, J. B. von. 1823. *Sim. Vespert. Brasil.*, p. 21.

Type locality: Brazil. Restricted by Hershkovitz [5] to the Rio Onças, municipality of Campos, Rio de Janeiro, Brazil.

#### Plecturocebus new genus

urn:lsid:zoobank.org:act:1E86C672-5008-4DB6-8776-53595C157FEA

Type species.* Plecturocebus moloch* (Hoffmannsegg, 1807) Red-bellied titi

*Cebus moloch* Hoffmannsegg, G. von. 1807. *Mag. Ges. Naturf. Fr*., Berlin, 9: 97.

Etymology: “Plect-” comes from the Greek *plektos*, which means plaited or twisted. In Latin, *Plecto* and *plexus* refer to a braid, plait, or interweave. “Uro-” comes from the Greek word *oura*, which means “tail”. “Cebus” is from the Greek *kebos*, which means “a long-tailed monkey”. The name refers to the tail-twining behaviour of the Callicebinae. Titis, adults and juveniles, frequently intertwine their tails when they sit side-by-side; sometimes looped quite loosely, sometimes wound around very tightly, making several turns. The behaviour is affiliative [38].

**Fig. 7:**
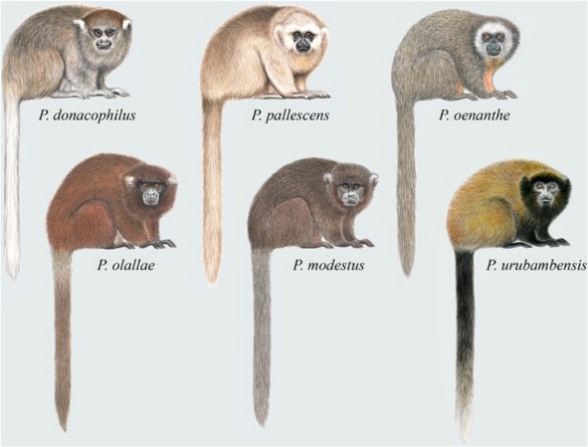
Titi monkeys, the *donacophilus* group of *Plecturocebus*. Illustrations by Stephen D. Nash ©Conservation International

**Fig. 8:**
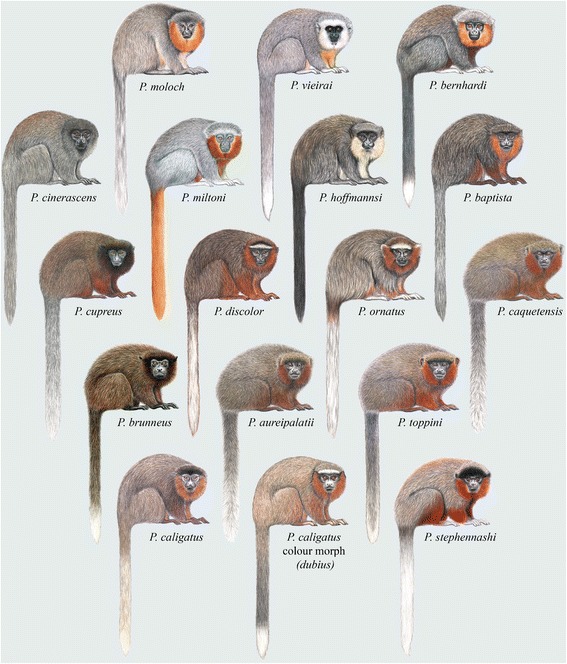
Titi monkeys, the *moloch* group of *Plecturocebus*. Illustrations by Stephen D. Nash ©Conservation International

Diagnostic characters: Hershkovitz’s [5] review contains detailed descriptions of the dental, cranial and post-cranial characters of the titi species recognized at the time, and presents summaries of the key characteristics of his *modestus* (included here in the *donacophilus* group), *donacophilus* (Fig. 7) and *moloch* (Fig. 8) groups. Groves’ [16] taxonomy, with some exceptions, followed that of Hershkovitz, and the distinguishing features he provided, and that we record here, are from Hershkovitz’s comprehensive 1990 review [5].

Groves [16] (p. 171) summarized the *modestus* group as follows: “Externally resembles the *moloch* group, but cranially primitive according to Hershkovitz [5], with an elongate, low-slung cranium, very small cranial capacity, only 20 % of greatest skull length, and short occiput, condylobasal length averaging 86 % of greatest skull length. Median pterygoids very large; mandibular angle large. Postcranial skeleton unknown; chromosomes unknown”.

Characteristics of species of the *donacophilus* group (*donacophilus*, *pallescens*, *olallae* and *oenanthe*) were summarized as follows by Groves [16] (p. 171): “Cranial capacity 21 − 25 % of greatest skull length, condylobasal length 81 − 84 % of greatest skull length. Arm (radius plus humerus) 52 − 58 % of trunk length, leg (tibia plus femur) 71 − 78 %. Chromosomes 2n = 50”.

Characteristics of the *moloch* group, including the species *cinerascens*, *hoffmannsi*, *baptista*, *moloch*, *brunneus*, *cupreus* (synonyms *caligatus*, *discolor*, *toppini*, and *dubius*), and *ornatus*, were summarized by Groves [16] (p. 172 − 173) as follows: “Cranial capacity 26 − 29 % of greatest skull length; condylobasal length 78 − 82 %. Forelimb (known only for *C. cupreus*) 53-61 % of trunk length, hindlimb 72 − 81 %. Chromosomes 2n = 48 (*C. moloch*, *C. brunneus*) or 46 (*C. cupreus*, *C. ornatus*)”. The groups began to diversify *c.* 4.39 Ma, in the Early Pliocene.

Geographic range: Brazil, Colombia, Ecuador, Peru, Bolivia, Paraguay (Fig. 5). The northernmost limit is the upper reaches of the Río Meta in Colombia (*Plecturocebus ornatus*) extending south to the upper Río Guaviare. *Plecturocebus caquetensis* occurs in a small portion of the upper Caquetá basin in Colombia. All other representatives of this genus occur throughout the greater part of the Amazon basin, south of the ríos Iça-Putumayo and Amazonas-Solimões, east of the Andes, extending south through Ecuador, Peru, Brazil, and Bolivia into Paraguay to the confluence of the ríos Pilcomayo and Paraguai. In Brazil, they occur east as far as the Rio Tocantins-Araguaia, south of the Rio Amazonas [4–8, 35, 39].

#### *Plecturocebus donacophilus* group

*Plecturocebus donacophilus* (D’Orbigny, 1836). White-eared titi

*Callithrix donacophilus* D’Orbigny, M. A. D. 1836. *Voy. Am. Merid., Atlas Zool*., pl. 5.

Type locality: Rio Mamoré basin, Beni, Bolivia.

*Plecturocebus pallescens* (Thomas, 1907). White-coated titi

*Callicebus pallescens* Thomas, O. 1907. *Ann. Mag. Nat. Hist*., 7^th^ ser., 20: 161.

Type locality: Thirty miles north of Concepción, Chaco, Paraguay.

*Plecturocebus oenanthe* (Thomas, 1924). Río Mayo titi

*Callicebus oenanthe* Thomas, O. 1924. *Ann. Mag. Nat. Hist*. 9^th^ ser., 14: 286.

Type locality: Moyobamba, San Martín, Peru, altitude *c.* 840 m above sea level.

*Plecturocebus olallae* (Lönnberg, 1939). Olalla Brother’s titi

*Callicebus olallae* Lönnberg, E. 1939. *Ark. f. Zool.,* 31A, 13: 16.

Type locality: La Laguna, 5 km from Santa Rosa, Beni, Bolivia, altitude *c.* 200 m above sea level.

*Plecturocebus modestus* (Lönnberg, 1939). Rio Beni titi

*Callicebus modestus* Lönnberg, E. 1939. *Ark. f. Zool.,* 31A, 13: 17.

Type locality: El Consuelo, Río Beni, Beni, Bolivia, altitude 196 m above sea level.

*Plecturocebus urubambensis* (Vermeer & Tello-Alvorado, 2015). Urubamba brown titi

*Callicebus urubambensis* Vermeer, J. & Tello-Alvorado, J. C. 2015. *Primate Conserv.* (29): 19.

Type locality: Peru: near the Colonia Penal del Sepa, on the southern bank of the Río Sepa, a western tributary of the Río Urubamba (10°48'50"S, 73°17'80"W). Altitude 280 m.

#### *Plecturocebus moloch* group

*Plecturocebus moloch* (Hoffmannsegg, 1807) Red-bellied titi

*Cebus moloch* Hoffmannsegg, G. von. 1807. *Mag. Ges. Naturf. Fr*., Berlin, 9: 97.

Type locality: Near the town of Belém, Pará, Brazil. Hill [26] gives the type locality as the banks of the Rio Pará (= terminal part of the Rio Tocantins), Pará, Brazil. Redetermined by Hershkovitz [15] as the right bank of the lower Rio Tapajós, municipality of Santarém, Pará, Brazil.

*Plecturocebus vieirai* (Gualda-Barros, Nascimento & Amaral, 2012). Vieira’s titi

*Callicebus vieirai* Gualda-Barros, J., Nascimento, F. O. do & Amaral, M. K. do. 2012. *Pap. Avuls. Zool., São Paulo* 52(53): 263.

Type locality: Rio Renato, tributary of Rio Teles Pires (right bank), nearby the city of Cláudia, state of Mato Grosso Brazil (11°33'00.15"S, 55°10'59.98"W); *c*. 370 m above sea level.

*Plecturocebus bernhardi* (M. G. M. van Roosmalen, T. van Roosmalen & Mittermeier, 2002). Prince Bernhard’s titi

*Callicebus bernhardi* Van Roosmalen, M. G. M., Van Roosmalen, T. and Mittermeier, R. A. 2002. *Neotrop. Primates* 10(suppl.): 24.

Type locality: West bank of the lower Rio Aripuanã, at the edge of the settlement of Nova Olinda, 41 km southwest of the town of Novo Aripuanã, Amazonas state, Brazil. 05°30'63"S, 60°24'61"W, altitude 45 m above sea level.

*Plecturocebus cinerascens* (Spix, 1823). Ashy titi

*Callithrix cinerascens* Spix, J. B. von. 1823. *Sim. Vespert. Brasil*., p. 20, pl.14.

Type locality: Unknown. Spix indicated the Río Putumayo-Içá in the vicinity of the Peru-Brazil border, but, as indicated by Hershkovitz [5], there is no evidence that it was ever collected there. This species occurs on right bank of the Rio Aripuanã, a tributary of the Rio Madeira, and on the both banks of the Rio Aripuanã above its confluence with the Rio Roosevelt [6, 40].

*Plecturocebus miltoni* (Dalponte, Silva & Silva-Júnior, 2014). Milton's titi

*Plecturocebus miltoni* Dalponte, J. C., Silva, F. E. & Silva-Júnior, J. de S. 2014. *Pap. Avuls. Zool., São Paulo* 54(32): 462.

Type locality: Curva do Cotovelo (08°59'45.21"S, 60°43'42.72"W), region of the mouth of the Pombal stream, Reserva Extrativista Guariba-Roosevelt, right bank of the upper Roosevelt River, municipality of Colniza, Mato Grosso, Brazil.

*Plecturocebus hoffmannsi* (Thomas, 1908). Hoffmanns’s titi

*Callicebus hoffmannsi* Thomas, O. 1908. *Ann. Mag. Nat. Hist*., 8^th^ series, 2: 89.

Type locality: Urucurituba, Santarém, Rio Tapajós, Pará, Brazil.

*Plecturocebus baptista* (Lönnberg, 1939). Lake Baptista titi

*Callicebus baptista* Lönnberg, E. 1939. *Ark. f. Zool.,* 31A, 13: 7.

Type locality: Determined by Hershkovitz [15] (p. 29) as the Lago do Baptista, right bank of the Rio Madeira, north of the Paraná Urariá and east of the town of Nova Olinda do Norte, Amazonas, Brazil [6]. Syntypes collected from the Lago Tapaiuna.

*Plecturocebus cupreus* (Spix, 1823) Coppery titi

*Callithrix cuprea* Spix, J. B. von. 1823. *Sim. Vespert. Brasil*., p. 23, pl. 17.

Type locality: Rio Solimões, Brazil, near the Peruvian boundary. Restricted to Tabatinga by Hershkovitz [15] (p. 36), but should be opposite Tabatinga because the species does not occur on the north bank or Tabatinga side of the Solimões [5] (p. 61).

*Plecturocebus discolor* (I. Geoffroy Saint-Hilaire & Deville, 1848). Red-crowned titi

*Callithrix discolor* Geoffroy Saint Hilaire, I. & Deville, É. 1848. *C. R. Acad. Sci. Paris*, 27: 498.

Type locality: Sarayacu, Río Ucayali, Ucayali, Peru.

*Plecturocebus ornatus* (Gray, 1866). Ornate titi

*Callithrix ornata* Gray J. E. 1866. *Ann. Mag. Nat. Hist*., 4^th^ ser., 17: 57.

Type locality: “Nouvelle Grenade”, now Colombia, restricted to the Villavicencio region, Río Meta, Meta, Colombia, by Hershkovitz [15] (p. 44).

*Plecturocebus caquetensis* (Defler, Bueno & Garcia, 2010). Caquetá titi

*Callicebus caquetensis* Defler, T. R., Bueno. M. L. & García, J. 2010. *Primate Conserv.* (25): 2.

Type locality: Vereda El Jardin, east of Valparaiso, municipality of Puerto Milan, Department of Caquetá, Colombia, 1°8'24.61"N, 75°32'34.04"W, 251 m above sea level.

*Plecturocebus brunneus* (Wagner, 1842). Brown titi

*Callithrix brunea* Wagner, J. A. 1842. *Arch. Naturgesch*., 8(1): 357.

Type locality: Brazil, subsequently specified by Pelzeln [41] (p. 20) as Rio Mamoré, Cachoeira da Bananeira, Rondônia, Brazil.

*Plecturocebus aureipalatii* (Wallace, Gómez, A. M. Felton & A. Felton, 2006). Madidi titi

*Callicebus aureipalatii* Wallace et al. 2006. *Primate Conserv.* (20): 31.

Type locality: Campamento Roco Roco, Río Hondo, Madid National Park and Natural Area of Integrated Management, La Paz Department, Bolivia (14°37'30"S, 67°43'06"W).

*Plecturocebus toppini* (Thomas, 1914). Toppin’s titi

*Callicebus toppini* Thomas, O. 1914. *Ann. Mag. Nat. Hist.*, ser. 8, 13: 480.

Type locality: Rio Tahuamanu, northeast Peru [sic] near Bolivian boundary. About 12°20'S, 68°45'W. The Rio Tahuamanu and the Bolivian border are in fact in southeast Peru, not northeast; evidently a *lapsus calami*.

*Plecturocebus caligatus* (Wagner, 1842). Chestnut-bellied titi

*Callithrix caligata* Wagner, J. A. 1842. *Arch. Naturgesch*., 8(1): 357.

Type locality: Restricted by Thomas [42] (p. 90) to Borba, Rio Madeira, Amazonas Brazil.

*Plecturocebus dubius* (Hershkovitz, 1988). Doubtful titi

*Callicebus dubius* Hershkovitz, P. 1988. *Proc. Acad. Nat. Sci. Philadelphia* 140(1): 264.

Type locality: Said to be Lago de Aiapuá (= Ayapuá), west bank, lower Rio Purus, more likely on the east bank of the lower Rio Purus, probably opposite of the Lago do Aiapuá [5]. Röhe & Silva-Júnior [43] recorded that the species had crossed from the Mucuim-Ituxi interfluvium to the right bank of the Rio Mucium using a man-made bridge. Here considered a junior synonym of *P. caligatus*.

*Plecturocebus stephennashi* (M. G. M. van Roosmalen, T. van Roosmalen & Mittermeier, 2002). Stephen Nash’s titi

*Callicebus stephennashi* Van Roosmalen, M. G. M., Van Roosmalen, T. and Mittermeier, R. A. 2002. *Neotrop. Primates* 10(suppl.): 15.

Type locality: Unknown, holotype and paratypes said to be have been caught somewhere along the middle to upper Rio Purus, Amazonas, Brazil.

### Genus-level topology

Our proposal to divide *Callicebus* into three distinct genera gains support from previous molecular phylogenetic analyses (e.g., [1, 2, 44]). Our divergence-time estimates for the genus-level splits (*Cheracebus c.* 11 Ma; *Callicebus c.* 8.3 Ma), are comparable to those reported by Springer et al*.* [2] (*Cheracebus c.* 7.8 Ma; *Callicebus c.* 7.2 Ma) and Perelman et al. [1] (*Callicebus c*. 9.9 Ma). Based on phylogenomic evidence, Jameson Kiesling et al*.* [21] estimated the divergence time of *Callicebus* and *Plecturocebus* at 6.7 Ma, and noted that these two species groups required the designation of separate genera.

The phyletic groups proposed by Kobayashi [13] using cranial morphometrics correspond with the arrangement found using molecular evidence in the present study. Kobayashi [13] noted that the *torquatus* group (*Cheracebus*) and the *personatu*s group (*Callicebus*) presented a high degree of character differentiation, while the *donacophilus*, *moloch* and *cupreus* groups (*Plecturocebus*) were more closely related. In discordance with his proposal, we found support for the division of *Plecturocebus* into two, not three, species groups. The *donacophilus* group is indeed a distinct early diverging lineage but Kobayashi’s [13] *moloch* and *cupreus* groups are better described as a single group, which began diversifying *c.* 3.4 Ma. To account for paraphyly in the current group arrangement, we propose that all Amazonian titis of the *cupreus* and *moloch* groups (*sensu* Kobayashi, 1995) should be assigned to a single *moloch* group, conforming to the *moloch* group identified by Groves [16]. We argue that increased resolution of the species-level relationships among these species is required to justify erecting any additional species group.

Body size and pelage colouration also support our taxonomic hypothesis. The *moloch* species group of *Plecturocebus* is composed of medium-sized ‘typical’ titis characterised by the greyish or brownish dorsum with a contrasting whitish, orange or reddish belly (except *P. cinerascens* and *P. brunneus*; see Fig. 8) [12], while the *donacophilus* clade taxa are the smallest species, generally showing a buffy to dark grey pelage that lacks contrast (Fig. 7) [13]. The Atlantic forest *Callicebus* are distinguished by their large size and overall appearance (Fig. 6), distinct from other callicebine taxa (see [16]). Hershkovitz [12] indicated that *Cheracebus* species are larger than the species of *Plecturocebus*, but Groves [16] (p. 176) found that this was not borne out by the available measurements. They are distinguishable from all other titis, however, by their uniform dark reddish to blackish pelage with contrasting whitish throat collar (Fig. 4) and also their postcranial skeleton.

Our conclusions based on molecular evidence are further supported by karyological data. The subfamily Callicebinae presents extensive karyotypic variation that corresponds closely to the present genera derived from molecular and morphological data. *Cheracebus* is characterised by low chromosome number; 2n = 20 in *C. torquatus* [45] and *C. lucifer* [14], and 2n = 16 in *C. lugens*, the lowest diploid chromosome number known among all primates [46]. *Callicebus nigrifrons* and *C. personatus*, show intermediate chromosome numbers of 2n = 42 and 2n = 44, respectively [47]. *Plecturocebus* taxa have the highest chromosome numbers, ranging from 2n = 44 (*P. ornatus*) [48] to 2n = 50 (*P. hoffmannsi*, *P. donacophilus*) [49, 50].

Wood & Collard [51] argued that the designation of a genus should include “an ecological situation, or adaptive zone, that is different from that occupied by the species of another genus”. Our three genera satisfy these conditions with each having distinct geographic distributions (Fig. 5) and habitat preferences [10]. The Atlantic forest *Callicebus* are entirely extra-Amazonian and geographically well separated from all other callicebines. They are found in the Atlantic Forest region of eastern Brazil, as far south and west as the Tietê-Paraná-Parnaíba river system, and as far north as the Rio São Francisco [37]. This includes the range of *C. barbarabrownae*, which occupies the Caatinga biome of northeast Brazil.

*Cheracebus* is the northernmost genus, occurring in the Amazon Basin to the west of the rios Branco and Negro (north of the Rio Amazonas) and west of the Rio Madeira (south of the Rio Amazonas), with the geographic range of *C. lugens* extending north of the Rio Negro into Venezuela and Colombia [10]. In the southern part of their range, *Cheracebus* species are sympatric with species of the *moloch* group of *Plecturocebus*, which occur throughout the southern and western Amazon basin (Fig. 5). However, it is unlikely that this has resulted in extensive niche overlap. *Cheracebus* species prefer open-canopy forests, with tall trees and well-drained soils, and make use of higher levels of the canopy, whereas *moloch* group species occupy the dense understoreys of vegetation, thick with lianas [7], [52]. Where they are sympatric, it has been reported that *Cheracebus* species often inhabit areas of poor vegetation, outcompeted by the *moloch* group species for more favourable habitats [53, 54]. Although still little studied, *Cheracebus* and sympatric *Plecturocebus* undoubtedly have different dietary preferences, with *Cheracebus* species consuming more insects, seeds and tougher fruits, while the diets of the *moloch* group species contain more leaves [10, 55–58].

The range of the *donacophilus* group species of *Plecturocebus* extends far south of the Amazon basin and they have the most disjunct set of species distributions of the titi monkey clades. They occupy forest patches and gallery forests in the savannah floodplains of Bolivia, Paraguay and Brazil, with the range of *P. pallescens* extending into the Chaco scrublands and Pantanal swamps in Paraguay and Brazil [9, 10, 59, 60].

As we have sequence data for only one species of the *donacophilus* clade, we are limited in our ability to make novel inferences about this group. Although our estimated time of diversification for the *donacophilus* and *moloch* clades (4.4 Ma) is below the time-based classification criteria for genera of 11 to 7 Ma suggested by Goodman et al. [23], the morphological, molecular and ecological differences between these two groups may justify a new classification for taxa of the *donacophilus* clade, pending increased taxonomic sampling and sequence data.

For the taxa not included in this study we will continue to follow the arrangement proposed by Groves [17] (Table 1), with the exception of *P. modestus*. Only a single adult specimen has been collected to date. Hershkovitz [12, 15] noted the unusual elongated skull of *P. modestus* and regarded it as the most primitive titi monkey species. Because of this, he created the *modestus* group, a proposal followed by Groves [16, 17]. Kobayashi [13] moved *P. modestus* to the *donacophilus* group, but stated “the phylogenetic position of *P. modestus* is morphometrically debatable” (p.119) and that a sufficient number of samples need to be collected to clarify placement. Although new observations have been made in the wild [61], to date, no further adult *P. modestus* specimens have been collected and thus we follow Kobayashi [13] in maintaining *P. modestus* in the *donacophilus* group.

### Species-level topology

Our phylogenetic analyses showed strong support for most of the nodes in the Callicebinae phylogeny. At species-level, phylogenetic relationships among taxa of *Cheracebus* and *Callicebus* are identical in all analyses, however they varied among species of the *moloch* group of *Plecturocebus*.

Based on the analysis of museum specimens, Auricchio [20] suggested that the pelage colouration of *P. bernhardi* is consistent with polymorphic variation found in *P. moloch* specimens, and considered *P. bernhardi* as a junior synonym of *P. moloch*. He states that a mitochondrial phylogeny also supports the classification of all “*moloch*” phenotypes as polymorphic variants of the same species, including *P. bernhardi* and a specimen from the Alta Floresta region (likely *P.* cf. *moloch*). However, the molecular data and phylogenetic trees were not presented in the study. This classification is in conflict with the results from our molecular datasets, showing consistent support for three distinct taxa, with a sister-clade relationship between *P. bernhardi* and *P. moloch*/*P.* cf. *moloch*. Divergence time analyses date the split between *P. bernhardi* and *P. moloch*/*P.* cf. *moloch* at *c.* 1.7 Ma, representing one of the oldest speciation events within the *moloch* group and providing strong support for the validity of *P. bernhardi* as a distinct species. *Plecturocebus moloch* and *P.* cf. *moloch* are highly supported as distinct sister-taxa across all datasets, and divergence time analyses date the split at *c.* 1.1 Ma, comparable to other speciation times within the *moloch* group. Seven *P. moloch* specimens from three different localities (see Additional file 8) are included in this study, however, in contrast, the earliest diversification event within *P. moloch* is estimated at *c.* 0.4 Ma. The molecular evidence presented here provides support for the designation of *P.* cf. *moloch* as a valid species. This taxon occurs in the Alta Floresta region of Mato Grosso, Brazil, and our group is currently working on this new species description (Boubli et al. in prep.).

Our results suggest that *P. caligatus* and *P. dubius* are geographical variants of the same polymorphic species. For the nuclear and combined datasets, *P. dubius* is a minimally diverged sister taxon of *P. caligatus* (estimated divergence time 0.5 Ma), and for the mitochondrial dataset, *P. dubius* is paraphyletic and most of the nodes within the *P. caligatus*/*P. dubius* clade show low support. The genetic distance values estimated for the cytochrome *b* locus between *P. caligatus* and *P. dubius* (see Additional file 5; 0.01–0.06) strongly suggest that *P. dubius* should be considered a geographical variant of *P. caligatus. Plecturocebus caligatus* occurs in the interfluve delineated by the rios Purús/Solimões/Madeira/Ipixuna, and to the southwest *P. dubius* is found between the rios Purús/Mucuím/Madeira (southern limit unknown). The pelage colouration of *P. caligatus* and *P. dubius* is also highly similar; Hershkovitz [12] noted that the only distinguishing feature between *P. caligatus* and *P. dubius* was the whitish frontal band found in the latter, and suggested that rather than indicating two distinct species, forehead colouration could be a variable feature in *P. caligatus*. Considering the morphological, molecular, and geographical affinities between *P. caligatus* and *P. dubius*, we propose the designation of *P. dubius* [5] as a junior synonym of a polymorphic *P. caligatus*. We suggest that the phenotypic differences found between these taxa represent geographic variation in pelage colouration.

Based on cranial morphometrics, Kobayashi [13] suggested that *P. brunneus* was closely related to his *moloch* group species, however, the skulls of *P. brunneus* studied were of two species, *P. urubambensis* and *P. brunneus*, which may have affected the results. Our analyses support a western Amazonian species-complex composed of *P. brunneus*, and Kobayashi’s *cupreus* group species, *P. cupreus* and *P. caligatus. Plecturocebus cupreus* is the earliest diverging lineage within this clade, and *P. brunneus* is the sister taxon to *P. caligatus* (*P. dubius*), in discordance with Groves’ proposal that *P. caligatus* and *P. dubius* were junior synonyms of *P. cupreus* [16]*.* Although the relationships between these west-Amazonian species are well resolved in the mitochondrial and combined datasets, the nuclear topology differs but with low support across most of the nodes. We consistently find two distinct *P. cupreus* clades, with an estimated divergence time of 1 Ma. These two clades are not sister in the nuclear dataset phylogenies. The *P. cupreus* clade A samples are from museum specimens with known localities in the Amazon basin, whereas those of *P. cupreus* clade B come from a collection of blood samples with no available skins, skulls or geographical data.

*Plecturocebus cinerascens* has an overall grey agouti pelage, lacking the contrasting colours characteristic of the *moloch* group, leading Hershkovitz [12] to suggest that *P. cinerascens* is the most primitive member. In this study, the nuclear dataset supports *P. cinerascens* as the earliest diverging lineage, forming a sister-clade to all other species of the *moloch* group, followed by the divergence of *P. hoffmannsi* and then *P. miltoni* and the rest of the moloch group. However, the mitochondrial dataset supports an alternative topology where *P. cinerascens* and *P. miltoni* form a sister-group to the *P. bernhardi* and *P. moloch* clade. Analyses based on combined data show the same topology as mitochondrial phylogenies, but with low support for the *P. cinerascens*/*P. miltoni* and *P. bernhardi*/*P. moloch* sister-group relationship, likely as a result of strong conflict between the nuclear and mitochondrial phylogenetic signals. Using mitochondrial loci alone does not resolve the phylogenetic position of *P. hoffmannsi*; however, the combined phylogenetic signal from nuclear and mitochondrial markers supports *P. hoffmannsi* as an early diverging lineage estimated at *c.* 3.44 Ma. All taxonomic reviews to date infer a close relationship with *P. baptista*, and thus our results suggest that *P. hoffmannsi* and *P. baptista* are a sister-clade to all remaining *moloch* group taxa, with the exception of *P. cinerascens* and *P. miltoni* (position unresolved).

The *P. caligatus* and *P. moloch* specimens sequenced by Perelman et al. [1] were incorrectly identified and our results indicate that their *P. caligatus* sample is *P. donacophilus*. The identity of the *P. moloch* specimen of Perelman et al. [1] is unknown; however, it is sister to our *P. hoffmannsi* individuals in all analyses and so we labelled it *P.* cf. *hoffmannsi*. In our divergence time analyses, we estimate that these taxa diverged *c.* 1.2 Ma, thus it is likely that *P.* cf. *hoffmannsi* is a distinct species. Further investigation is required to confirm whether *P.* cf. *hoffmannsi* is one of the known species of *Plecturocebus* that have not been analysed, or a new taxon. It is also possible both these specimens from Perelman et al. [1] are captive hybrids.

### Divergence-time estimation and biogeography

Our time-calibrated phylogeny suggests that the callicebine lineages began to radiate in the Late Miocene, with the origin of *Cheracebus* at around 11 Ma, followed by the divergence of *Callicebus* and *Plecturocebus* at around 8.3 Ma. The timescale for titi monkey evolution estimated here is compatible with the fossil record of the platyrrhines and with other recent molecular analyses (see Table 3) [1–3, 21]. Within *Plecturocebus*, we find evidence for deeply divergent lineages leading to *P. donacophilus*, *P. hoffmannsi*, and the remaining taxa that date to the Pliocene, *c.* 4.4 Ma and 3.4 Ma, respectively. Within the *moloch* group, we find a sister-clade relationship between east and west-distributed Amazonian species, which diverged *c*. 2.8 Ma. Nearly all the *moloch* group sister-species divergences in this study occurred 2–1 Ma, pointing to a rapid Pleistocene diversification of this group.

**Table 3:** Comparison of estimated divergence times (combined dataset) with other recent studies

Clade or Split	Mean age (Ma)				
	Perelman *et al.* [1]	Springer *et al*. [2]	Schrago *et al*. [3]	Kiesling *et al.* [21]	Present study
Crown Pitheciidae	24.82	23.3	21.9	25.51	21.47
Pitheciinae vs. Callicebinae	20.24	20.7	19.6	18.08	18.71
*Cheracebus* vs. *Callicebus + Plecturocebus*	n/a	7.81	n/a	n/a	10.98
*Callicebus* vs. *Plecturocebus*	9.86	7.16	n/a	6.65	8.34
*Plecturocebus: donacophilus* group vs. *moloch* group	4.69	3.22	n/a	n/a	4.39

*Abbreviations: Ma* millions of years ago, *n/a* not available

The three callicebine genera we propose here are isolated from each other by major biogeographical barriers: the Amazonian *Plecturocebus* titis are largely separated from the northernmost genus, *Cheracebus*, by the Rio Amazonas, and from the Atlantic Forest genus, *Callicebus*, by the Cerrado and Caatinga biomes of central Brazil (Fig. 5). At the species level, larger rivers in Amazonia frequently delimit the geographic distribution of titi monkeys, and recent evidence suggests that they can act as isolating barriers for sister taxa, promoting vicariant divergence [62]. Together, these characteristics make the subfamily Callicebinae of particular interest for the study of Amazonian biogeographical history.

## Conclusions

In this study, we provide the first molecular review of the subfamily Callicebinae, and our phylogenetic analyses help to clarify a number of issues on the taxonomic relationships among its species and genera. We provide evidence for an early divergence of three major callicebine lineages, and infer a highly supported phylogeny for all species included, with the exception of *P. miltoni* and *P. cinerascens*, which require further investigation. We support the reintegration of *cupreus* group species (*sensu* Kobayashi, 1995) into the *moloch* group and propose the designation of *P. dubius* as a junior synonym of *P. caligatus*.

The three callicebine genera identified here can be clearly separated on biogeographical, morphological and molecular grounds, and together, these factors provide strong evidence in support of our taxonomic proposal. Recent taxonomic revisions using molecular, ecological and morphological evidence have argued for the separation at the generic level of the robust and the gracile capuchins [63] and, likewise, saddleback and black-mantled tamarins from the remaining species of the genus *Saguinus* [64]. As with the tamarins and capuchins, this new classification will undoubtedly make for a taxonomy that reflects more clearly titi monkey evolutionary history. The lack of available genetic data for many of the species, however, limits our ability to make novel taxonomic and phylogenetic inferences about these taxa. It is evident that questions remain regarding the species-level taxonomy of the Callicebinae, and thus phylogenetic hypotheses will be modified with the availability of sequence data for remaining titi species. Taken together, our work illustrates the value of a molecular phylogenetic approach to taxonomic classification and here provides a basis for future studies on the evolutionary history and taxonomy of titi monkeys*.*

## Methods

### Taxon sampling

A total of 50 fresh tissue samples were collected from museum voucher specimens from the following Brazilian institutions: National Institute of Amazonian Research (INPA), Federal University of Pará (UFPA), Federal University of Rondônia (UNIR), Federal University of Amazonas (UFAM) and the Goeldi Museum (MPEG). The majority of these specimens were obtained in the context of an Amazonian-wide faunal inventory project (CNPq/SISBIOTA) carried out in accordance with the appropriate collection permits (IBAMA 483 license No. 005/2005 – CGFAU/LIC). This research adhered to the American Society of Primatologists’ and American Society of Mammalogists' principles for the ethical treatment of primates, and Brazilian laws that govern primate research.

Fifteen species of *Callicebus* were sampled, including representatives from each of the species groups of Kobayashi [13], and five platyrrhine species were selected as outgroup taxa. A complete list of *Callicebus* and outgroup species is presented in Additional file 8. We generated novel sequence data for a total of 49 *Callicebus* and 1 outgroup sample (JPB100, *Cebus albifrons*). All samples used in this study were from wild specimens, nearly all of which are of known provenance, and morphologically identified following Hershkovitz [5, 12], Van Roosmalen et al. [6], and Dalponte et al*.* [65]. Three of these samples are from a new species of *Callicebus* from the Alta Floresta region of Mato Grosso, Brazil (Boubli et al., in prep), that is closely related to *C. moloch* based on geographic location and pelage colouration, and is classified here as *C.* cf. *moloch*.

We retrieved additional sequences from GenBank representing six *Callicebus* and five outgroup samples from Perelman et al. [1], and another four *Callicebus* and four outgroup individuals. A total of 59 *Callicebus* and 10 outgroup individuals were included in this study. Additional information for all samples is presented in Additional file 8.

Of the six *Callicebus* specimens retrieved from the Perelman et al. [1] study, our molecular datasets confirm the taxonomic validity of *C. nigrifrons* (CNI-1) and show that *C. moloch* (CMH-1) and *C. caligatus* (CCG-1) are incorrectly identified. The *C. moloch* (CMH-1) specimen is most similar to our *C. hoffmannsi* individuals and *C. caligatus* (CCG-1) is very closely related to their *C. donacophilus* specimen (CDO-1). These samples are classified as *C.* cf. *hoffmannsi* and *C. donacophilus*, respectively (Additional file 8), but we note that these samples are of captive origin and could be captive hybrids.

### Molecular dataset

DNA sequence data were obtained from a total of 22 loci. We selected primers for 20 independent nuclear loci from Perelman et al. [1] based on their performance for *Callicebus*. Most of these primers were designed for the Perelman et al. [1] study, but some originated in previous studies [66–69]. The nuclear regions included exons, introns, and 3’UTRs, and two loci located on the X chromosome. We also obtained DNA sequence data from two mitochondrial loci; we amplified the cytochrome *b* gene (CYTB) with novel primers designed for this study (by JCC), and cytochrome *c* oxidase I (COI) using previously designed primers [70]. A complete list of loci and information on primers are presented in Additional file 9.

A total of 944 new sequences (nuclear and mitochondrial) were generated for this study from three laboratories: Universidade Federal do Pará (UFPA), Pará, Brazil; University of Salford, Manchester, UK; and the Evolution and Animal Genetics Laboratory (LEGAL), Universidade Federal do Amazonas (UFAM), Amazonas, Brazil. All new sequences were deposited in GenBank under the accession numbers presented in Additional file 10. We retrieved an additional 209 nuclear sequences for the 11 individuals sequenced for Perelman et al. [1] from GenBank, and 12 mitochondrial sequences from GenBank (Additional file 10).

Three datasets were compiled from subsets of loci and samples (Table 4): the nuclear dataset composed of the 20 nuclear loci totalling 12,778 bp in length; the combined dataset including all 22 loci totalling 14,578 bp in length; and the mitochondrial dataset composed of the two mitochondrial loci and a length of 1,800 bp. A summary of each dataset is presented in Table 4. The nuclear and combined datasets were composed of the same set of samples, containing 47 *Callicebus* and one outgroup sequenced for this study, and the 6 *Callicebus* and 5 outgroup individuals from Perelman et al. [1]. The mitochondrial dataset included all 50 newly sequenced samples, as well as an additional eight individuals retrieved from GenBank. A list of samples and number of loci sequenced for the nuclear and combined datasets is presented in Additional file 11 and for the mitochondrial dataset in Additional file 12. All *Callicebus* and outgroup species are represented in each dataset, with the exception *C.* cf. *hoffmannsi* and *C. coimbrai* (nuclear and combined only).

**Table 4:** Summary of dataset characteristics and sequence variation for *Callicebus* taxa

Dataset ID	Dataset description	Length (bp)	Missing data (%)	Constant sites		Variable sites		Parsimony informative sites		*Callicebus* samples
				bp	% of total	bp	% of total	bp	% of total	
Nuclear	20 nuclear loci^a^	12,778	13.6	12,387	96.9	391	3.1	293	2.3	53 samples; 47 sequenced for this study, 6 for Perelman *et al*. [1]
Combined	22 loci: 20 nuclear^a^, COI and CYTB	14,578	14.6	13,735	94.2	843	5.8	678	4.7	
Mitochondrial	2 mitochondrial loci: COI and CYTB	1,800	7.1	1,312	72.9	488	27.1	420	23.3	53 samples; 49 sequenced for this study, 4 from GenBank

*Abbreviations: COI* cytochrome *c* oxidase I, *CYTB* cytochrome *b*

^a^See Additional file 9 for a list of nuclear loci

For all datasets, *Callicebus* sample coverage for individual gene regions varied from 74 % to 100 % (average sample coverage = 90 %). Length of loci varied between 402 bp and 1140 bp. A list of loci characteristics is presented in Additional file 13.

### DNA isolation, amplification and sequencing

DNA was extracted from multiple tissues (blood, muscle, kidney) using the Promega Wizard Genomic Kit according to the manufacturer's protocol. We amplified all nuclear and mitochondrial gene regions using polymerase chain reaction (PCR). The PCR reactions were carried out in a total volume of 25 μL, containing approximately 30 ng of genomic DNA; 4 μL of dNTPs (1.25 mM); 2.5 μL 10X buffer (200 mM Tris-HCL, 500 mM KCl); 1 μL of MgCl_2_ (25 mM); 0.2 μM of each forward and reverse primer; and 1 Unit of Invitrogen™ *taq* DNA polymerase. The amplification cycles were carried out under the following conditions; initial denaturation at 95 °C for 5 min; followed by 35 cycles of denaturing at 94 °C for 1 min, primer annealing at between 44 °C and 64 °C (temperature varies per primer, see Additional file 9) for 1 min, and extension at 72 °C for 1 min; a final extension was carried out at 72 °C for 5 min.

PCR products were analysed on 1.5 % agarose gels and those that produced clear single bands were purified with polyethylene glycol (PEG) and ethanol [71]. After purification, PCR products were sequenced directly in two reactions with forward and reverse primers. Sequencing reactions were carried out using the BigDye Terminator v3.1 cycle sequencing kit (Life Technologies). For 10 μL sequencing reactions we used 0.5 μL of BigDye; 1.5 μL of 5X Sequencing buffer; 1.0 μL of each primer (0.8 μM); and 2 μL of PCR product. Sequencing reactions were performed as follows: 96 °C for 2 min; followed by 35 cycles of 96 °C for 15 s, 50 °C for 15 s, 60 °C for 2.5 min. The sequencing products were analysed using an ABI 3500xl (Life Technologies) automatic sequencer following the manufacturer’s instructions. Consensus sequences for each individual were generated from sequences in forward and reverse directions using Geneious R7.1 (Biomatters).

### Sequence alignment, data partitioning and model selection

Each locus was first aligned independently using the standard MUSCLE [72] alignment plugin in Geneious R7.1 and checked visually. The loci were then concatenated into alignments reflecting the three datasets (nuclear, combined and mitochondrial).

We used the program PartitionFinder [73] to objectively determine the optimal model of evolution and partitioning scheme simultaneously. Best-fit models were selected using Bayesian information criteria under a ‘greedy’ search scheme using a subset of models specific to each programme used (RAxML, MrBayes, BEAST). When specifying the alignment subsets for PartitionFinder, we defined all intronic and UTR loci as single data-blocks and split exonic sequences into three subsets reflecting codon position. All our phylogenetic analyses used a specific partitioning scheme (containing between 3 and 9 partitions) selected for the dataset by PartitionFinder. Additional information about each specific partitioning scheme is presented in Additional file 14.

### Phylogenetic analyses

We conducted phylogenetic inference using maximum-likelihood (ML) and Bayesian methods for each dataset. All phylogenetic analyses were run on the CIPRES Science Gateway v 3.3 server [74]. Our ML phylogenetic reconstructions were conducted using the program RAxML v. 8.1 [75]. For ML inferences, we used the partitioning scheme and best-fit models chosen by PartitionFinder. We estimated support for nodes using the rapid-bootstrapping algorithm (−f a -x option) for 1000 non-parametric bootstrap replicates [76]. Maximum-likelihood bootstrap support values (BP) greater than 70 % were considered strong support [77].

Bayesian analyses were performed using MrBayes 3.2.3 [78] with the Metropolis coupled Markov Chain Monte Carlo (MCMC) algorithm. The partitioning scheme and best-fit models chosen by PartitionFinder were implemented and partitions were unlinked. MCMC convergence was checked aſter two independent four-chain runs of 10 million generations for each Bayesian inference. We assessed convergence by examining LnL, the average standard deviation of the split frequencies between the two simultaneous runs (<0.01), and the Potential Scale Reduction Factor (PSRF) diagnostic in MrBayes, after a burn-in of 10 %. Posterior probability values (PP) higher than 0.95 were considered strong support [79].

A divergence matrix for the cytochrome *b* locus was generated for selected taxa (*C. cupreus*, *C. brunneus*, *C. caligatus*, *C. dubius*) using PAUP*4.0, based on the model parameters selected for the alignment by jModelTest v 2.1.6 [80, 81].

### Divergence-time analyses

We jointly estimated phylogeny and diversification times under an uncorrelated lognormal relaxed clock in the program BEAST v. 1.8.1 [82]. The partitioning scheme and best-fit models chosen by PartitionFinder were implemented and a Yule speciation process was used for all analyses. We ran two independent analyses for 50 million generations, sampling every 5000 generations. The sampling distributions of each run were visualized using Tracer v. 1.6 to evaluate convergence and to verify that the effective sample size was > 200 for all parameters after a burn-in of 10 %. We combined runs using LogCombiner v. 1.8.1 and generated the maximum credibility tree in TreeAnnotator v. 1.8.1.

To obtain the posterior distribution of the estimated divergence times, we used two calibration points with lognormal priors to set a hard minimum and soft maximum bound [83]. We set a minimum age of 15.7 Ma for crown Pitheciidae based on the fossil *Proteropithecia* Kay et al., 1998 [84], [85], and a minimum age of 12.5 Ma on crown Cebinae using the fossil *Neosaimiri* Stirton, 1951 [86–88]. For both calibration points, we set a soft maximum bound at 26 Ma using the fossil *Branisella boliviana* Hoffstetter, 1969, from the Deseadan fauna of La Salla [89]. We chose this maximum age based on the evidence that *Branisella boliviana* and the Miocene Patagonian fossils belong to independent stem platyrrhine radiations [3, 90, 91], the absence of fossils for extant lineages in South American formations from this period [91], and the wealth of molecular evidence in support of a more recent common ancestor for extant platyrrhines [2, 3, 92, 93]. The calibration points were implemented as lognormal distributions with an offset as the hard minimum bound. We set the standard deviation and mean such that 95 % of the prior distribution falls before the maximum age to create a soft maximum bound (Table 5).

**Table 5:** Evolutionary rate calibration constraints (in millions of years)

Divergence	Offset fossil	Offset	95 % age fossil	95 % prior distribution	Standard deviation	Mean	References
Pitheciinae/Callicebinae	*Proteropithecia*	15.7	*Branisella boliviana*	26	0.8	1.016	[84, 85, 89]
*Cebus*/*Saimiri*	*Neosaimiri*	12.5	*Branisella boliviana*	26	0.8	1.287	[86–89]

Our divergence-time analyses were run based on all 22 loci in the combined dataset, but to minimise missing data for these analyses, we concatenated sequences from two individuals for some outgroup species (*Cacajao calvus*, *Chiropotes israelita*, *Pithecia pithecia*, *Saimiri sciureus*; see Additional file 8). For comparison of node dates and topology, we also ran our BEAST analyses using the nuclear dataset.

### Nomenclatural Acts

The electronic edition of this article conforms to the requirements of the amended International Code of Zoological Nomenclature (ICZN), and hence the new names contained herein are available under that Code from the electronic edition of this article. This published work and the nomenclatural acts it contains have been registered in ZooBank, the online registration system for the ICZN. The ZooBank LSIDs (Life Science Identifiers) can be resolved and the associated information viewed through any standard web browser by appending the LSID to the prefix “http://zoobank.org/”. The LSID for this publication is: urn:lsid:zoobank.org:pub:A6DE1907-60DE-4968-BFE2-7964B13E02D8. The electronic edition of this work was published in a journal with an ISSN, and has been archived and is available from the following digital repositories: PubMed Central, LOCKSS, and University of Salford UK.

## Additional files

Additional file 1:
**Node support values and divergence time estimates for all phylogenetic analyses.** Node numbers correspond to those on Fig. 1, 2, 3, and Additional file 2, 3, 4, 6, 7. Bold indicates an unsupported node. Asterix indicates the node is not represented in that topology. (XLSX 38 kb) Additional file 2:
**Phylogenetic trees inferred from the combined dataset.** Shown are the phylogenetic trees with node support values based on maximum likelihood (A: RAxML) and Bayesian (B: MrBayes, C: BEAST) methods of analysis. Red numbers represent nodes of interest listed in Additional file 1. (PDF 4917 kb) Additional file 3:
**Phylogenetic trees inferred from the nuclear dataset.** Shown are the phylogenetic trees with node support values based on maximum likelihood (A: RAxML) and Bayesian (B: MrBayes, C: BEAST) methods of analysis. Red numbers represent nodes of interest listed in Additional file 1. (PDF 4875 kb) Additional file 4:
**Phylogenetic trees inferred from the mitochondrial dataset.** Shown are the phylogenetic trees with node support values based on maximum likelihood (A: RAxML) and Bayesian (B: MrBayes) methods of analysis. Red numbers represent nodes of interest listed in Additional file 1. (PDF 2750 kb) Additional file 5:
**Divergence matrix for the cytochrome** ***b*** **locus for selected** ***Callicebus*** **species.** Bold indicates distance values < 0.01. (XLSX 42 kb) Additional file 6:
**BEAST time-calibrated phylogeny inferred from the combined dataset.** Node bars indicate the 95 % highest posterior density. Red numbers represent nodes of interest listed in Additional file 1. (PDF 1903 kb) Additional file 7:
**BEAST time-calibrated phylogeny inferred from the nuclear dataset.** Node bars indicate the 95 % highest posterior density. Red numbers represent nodes of interest listed in Additional file 1. (PDF 1952 kb) Additional file 8:
**List of genetic samples used in this study including ID, source and corresponding dataset.** (XLSX 52 kb) Additional file 9:
**List of the 22 loci used in this study and primer information.** (XLSX 39 kb) Additional file 10:
**List of the GenBank accession numbers for all sequences.** In bold are the sequences newly generated for this study. (XLSX 58 kb) Additional file 11:
**List of sequence length and loci coverage for samples in the combined and nuclear datasets.** (XLSX 49 kb) Additional file 12:
**List of sequence length and loci coverage for samples in the mitochondrial dataset.** (XLSX 49 kb) Additional file 13:
**List of sequence characteristics per locus including length, variation, sample coverage and dataset.** Site information and sample coverage represent *Callicebus* taxa only. (XLSX 44 kb) Additional file 14:
**Partitioning schemes and substitution models selected by PartitionFinder.** The selected partitioning schemes were implemented in RAxML v. 8.1, MrBayes 3.2.3 or BEAST v 1.8.1. Numbers in parentheses refer to codon position for coding mitochondrial and nuclear sequences. (XLSX 28 kb)
