# Supplementary figures and images for: Phylogenetic relationships of the New World titi monkeys (Callicebus): first appraisal of taxonomy based on molecular evidence
Source: Front Zool. 2016 Mar 1;13:10. doi: 10.1186/s12983-016-0142-4 (PMC4774130; doi:10.1186/s12983-016-0142-4)

A

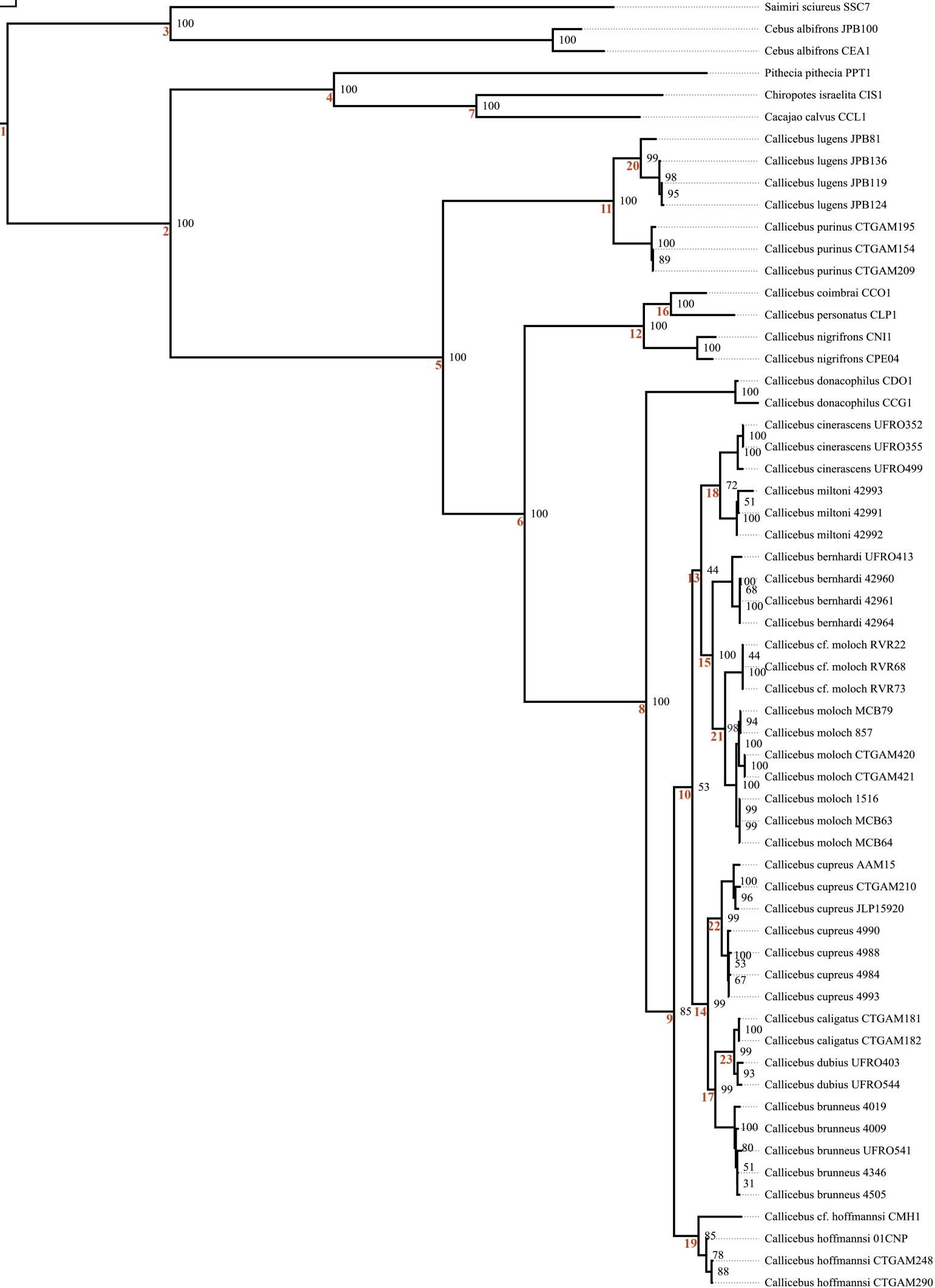

0.0050

B

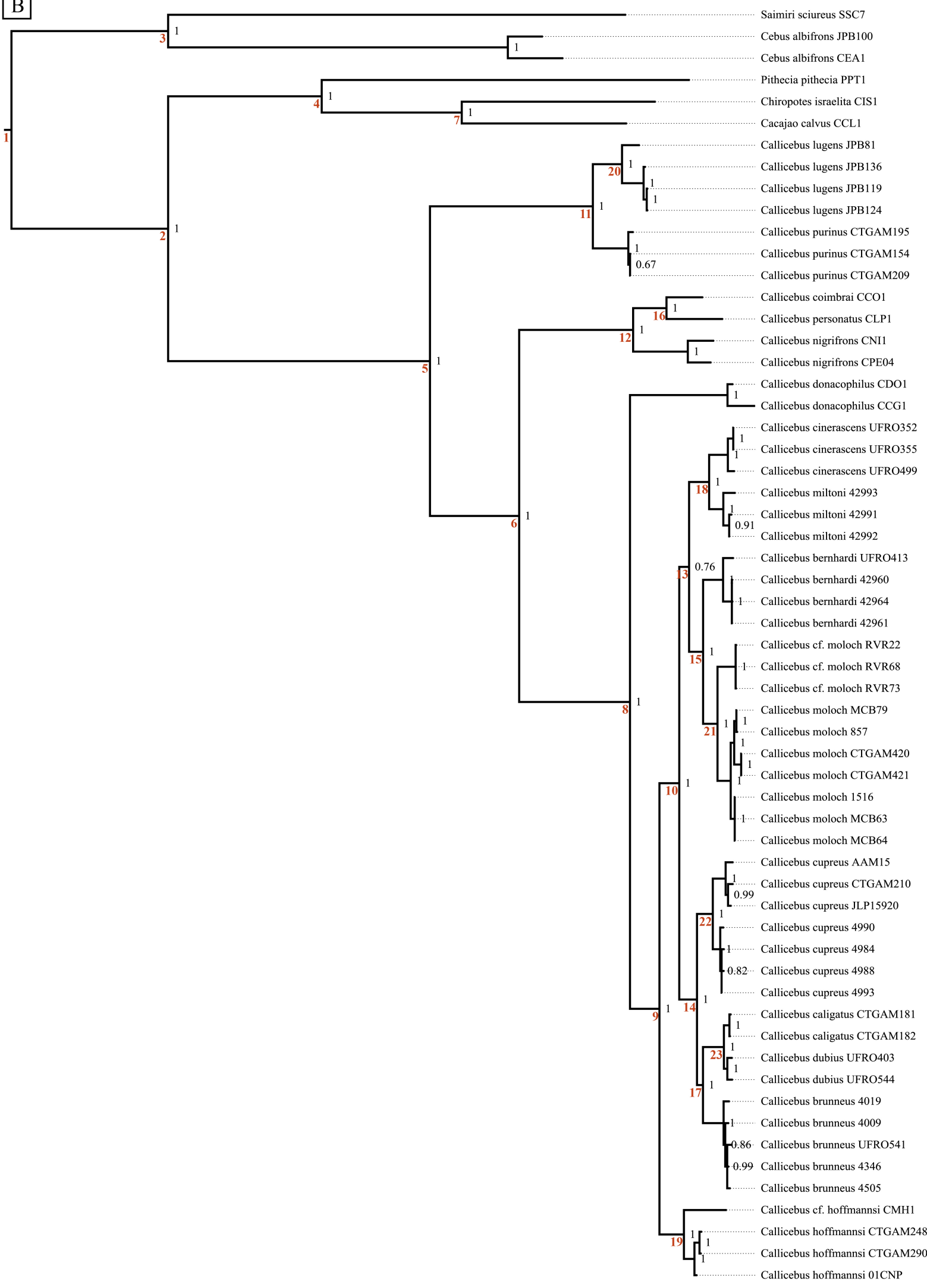

0.0050

C

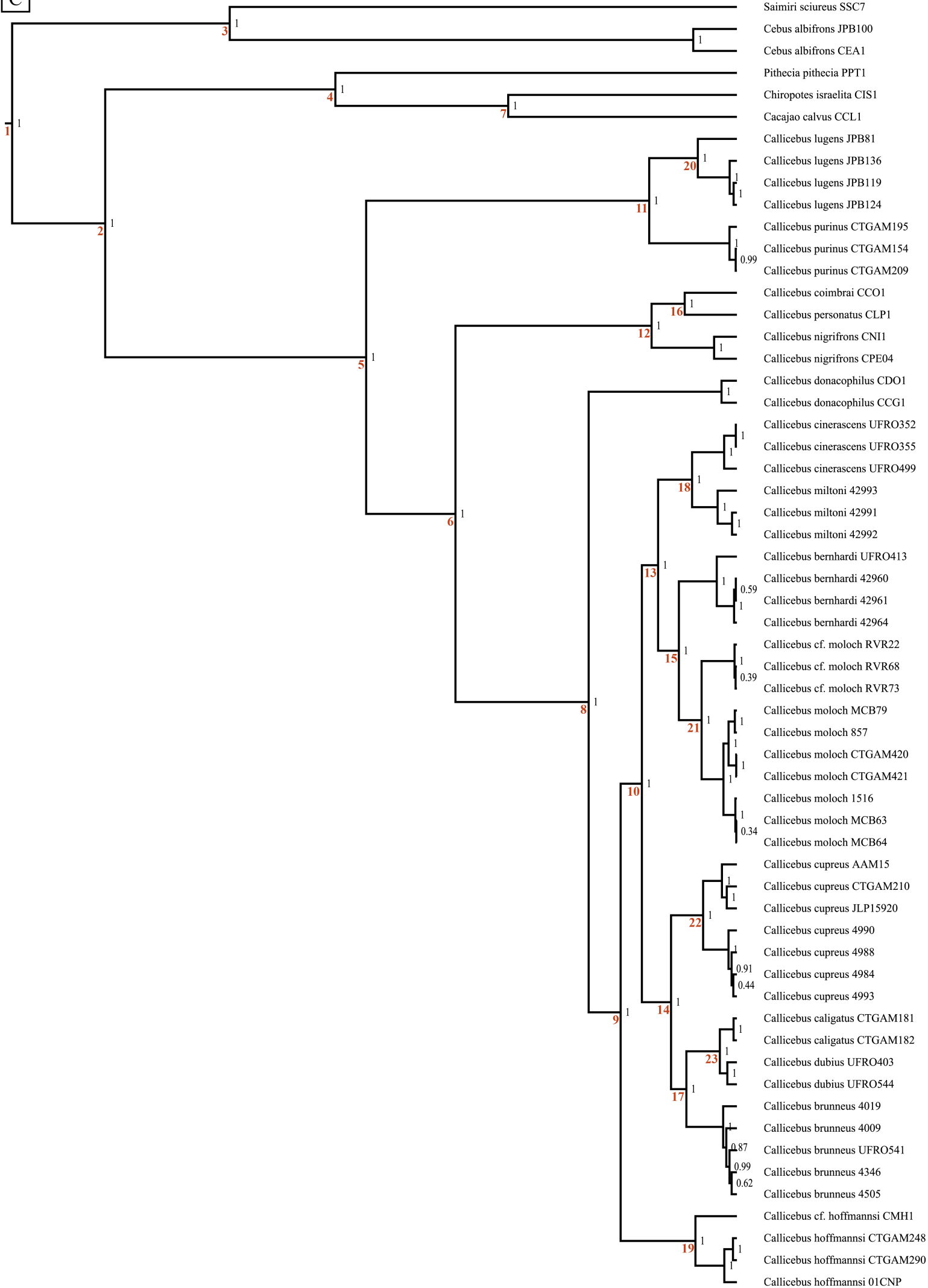

Supplement: Additional file 2: — Phylogenetic trees inferred from the combined dataset. Shown are the phylogenetic trees with node support values based on maximum likelihood (A: RAxML) and Bayesian (B: MrBayes, C: BEAST) methods of analysis. Red numbers represent nodes of interest listed in Additional file 1. (PDF 4917 kb) [file 12983_2016_142_MOESM2_ESM.pdf]

A

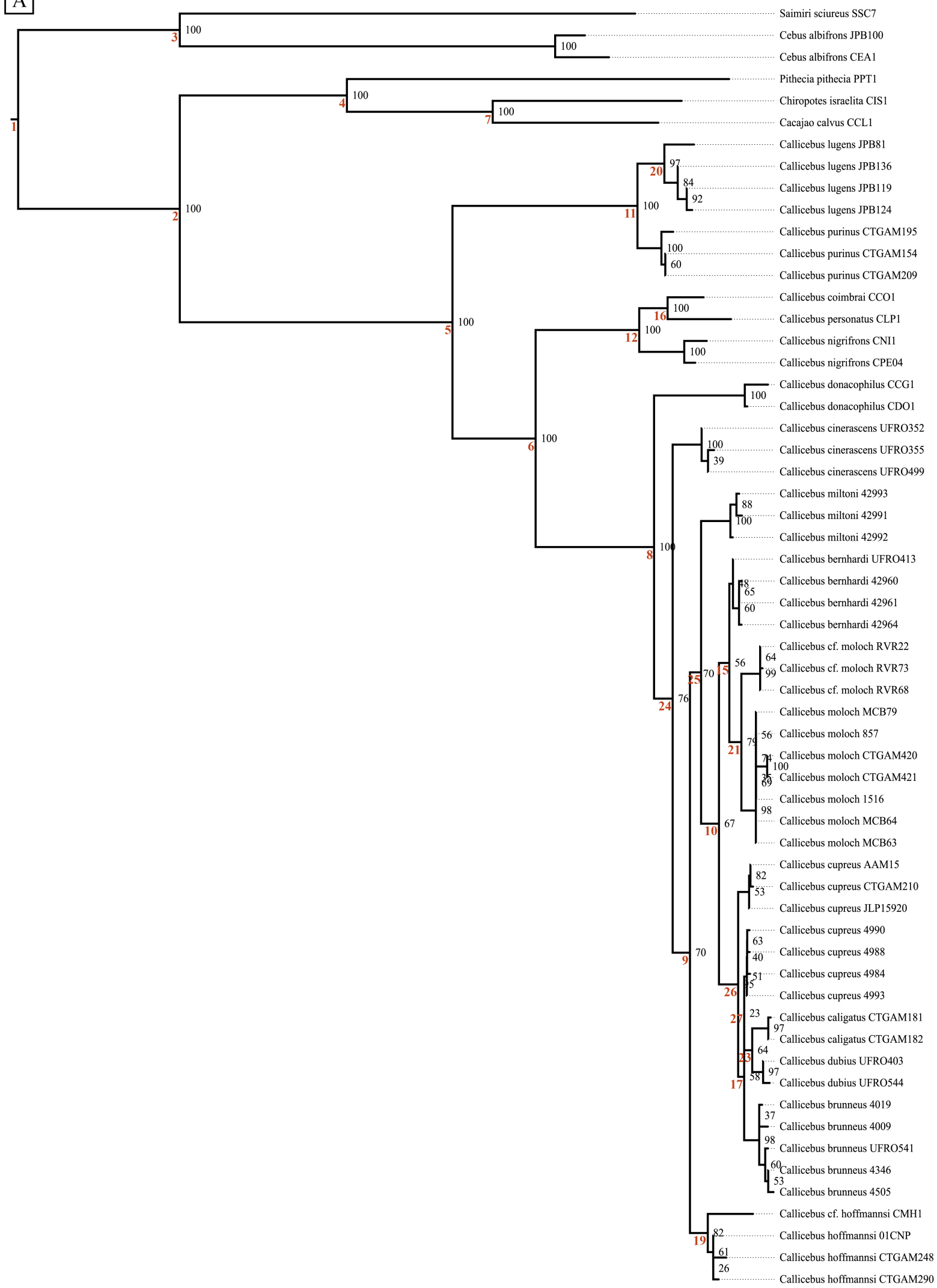

0.0050

B

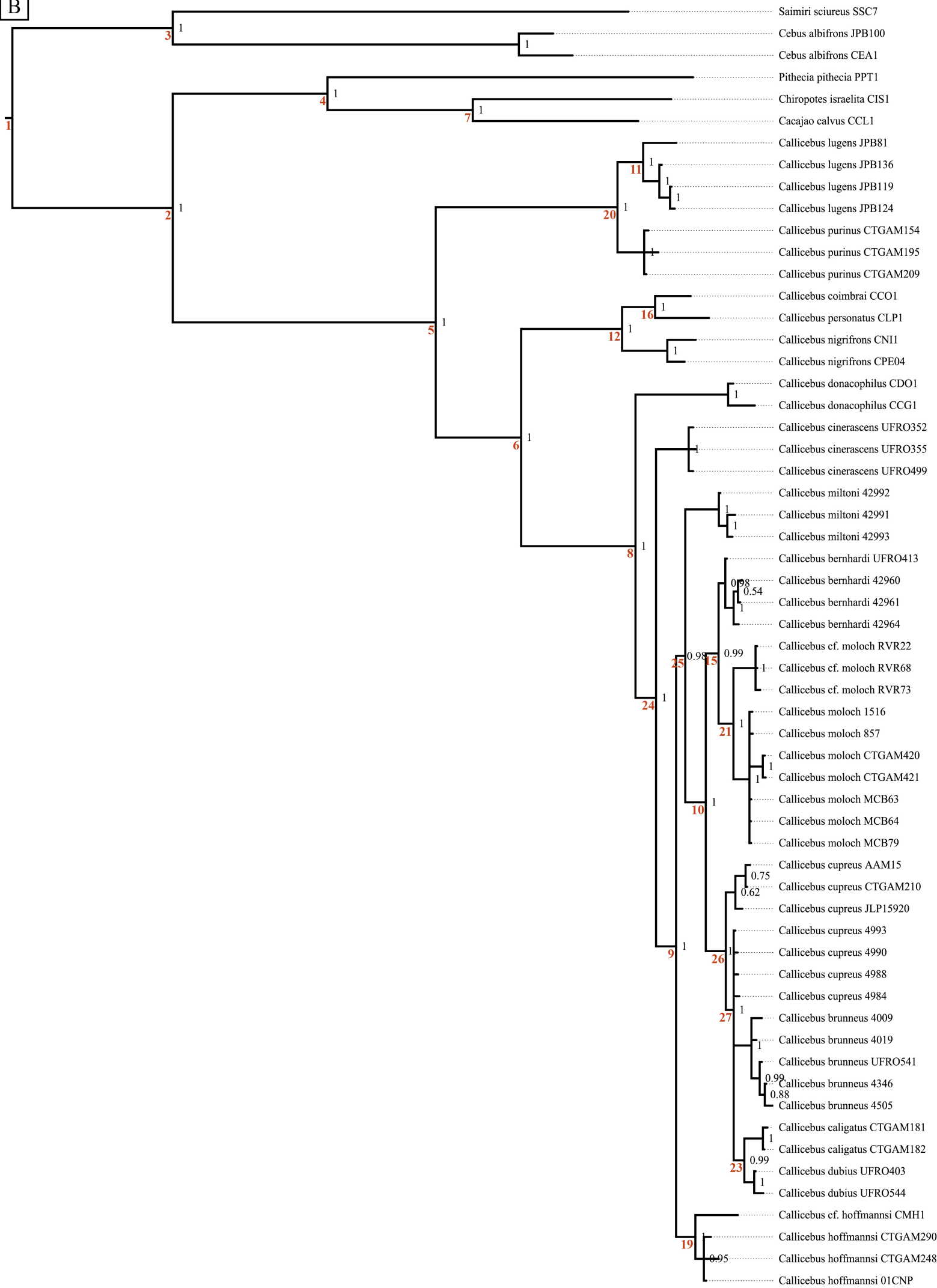

0.0050

C

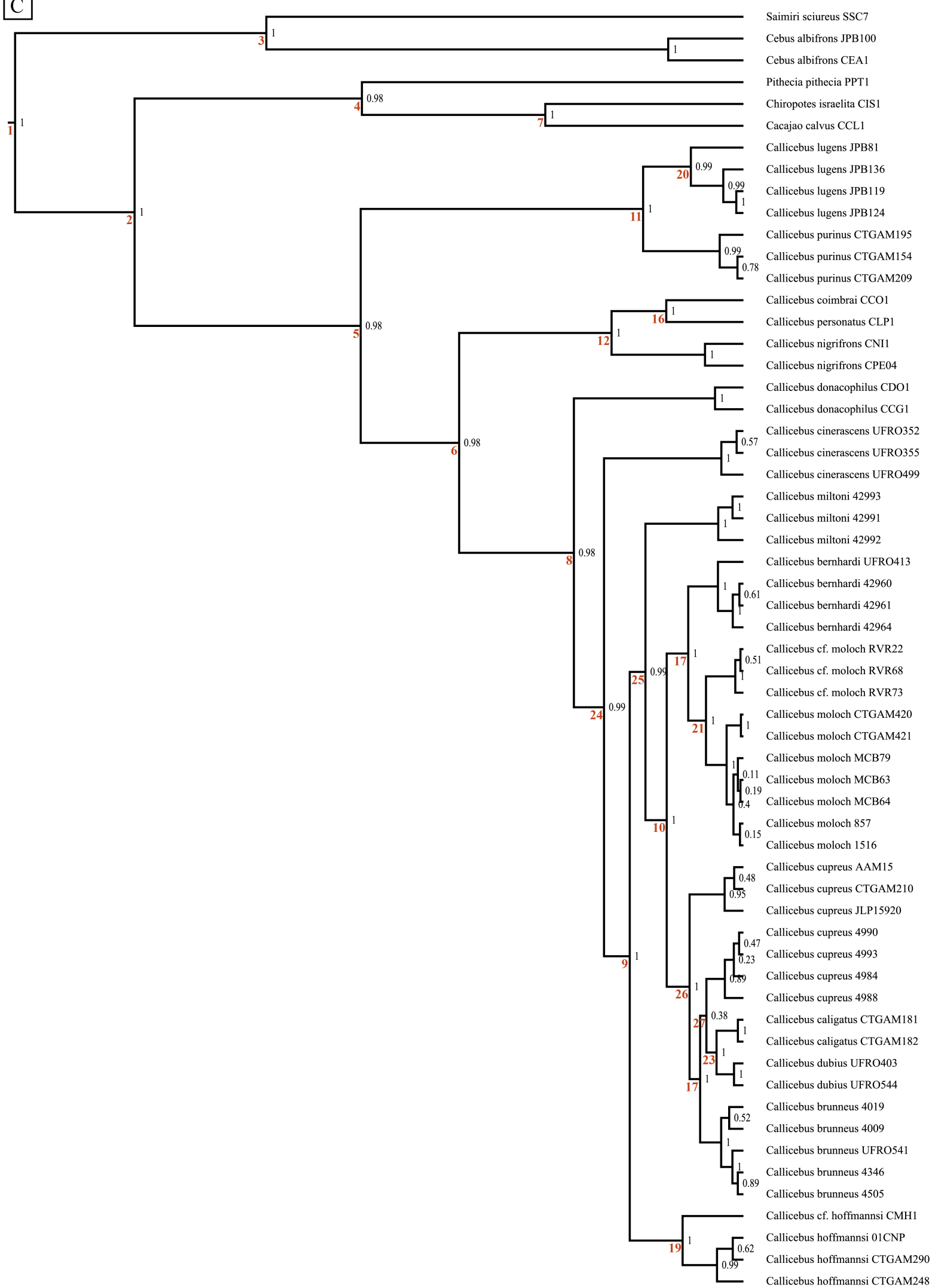

3.0

Supplement: Additional file 3: — Phylogenetic trees inferred from the nuclear dataset. Shown are the phylogenetic trees with node support values based on maximum likelihood (A: RAxML) and Bayesian (B: MrBayes, C: BEAST) methods of analysis. Red numbers represent nodes of interest listed in Additional file 1. (PDF 4875 kb) [file 12983_2016_142_MOESM3_ESM.pdf]

A

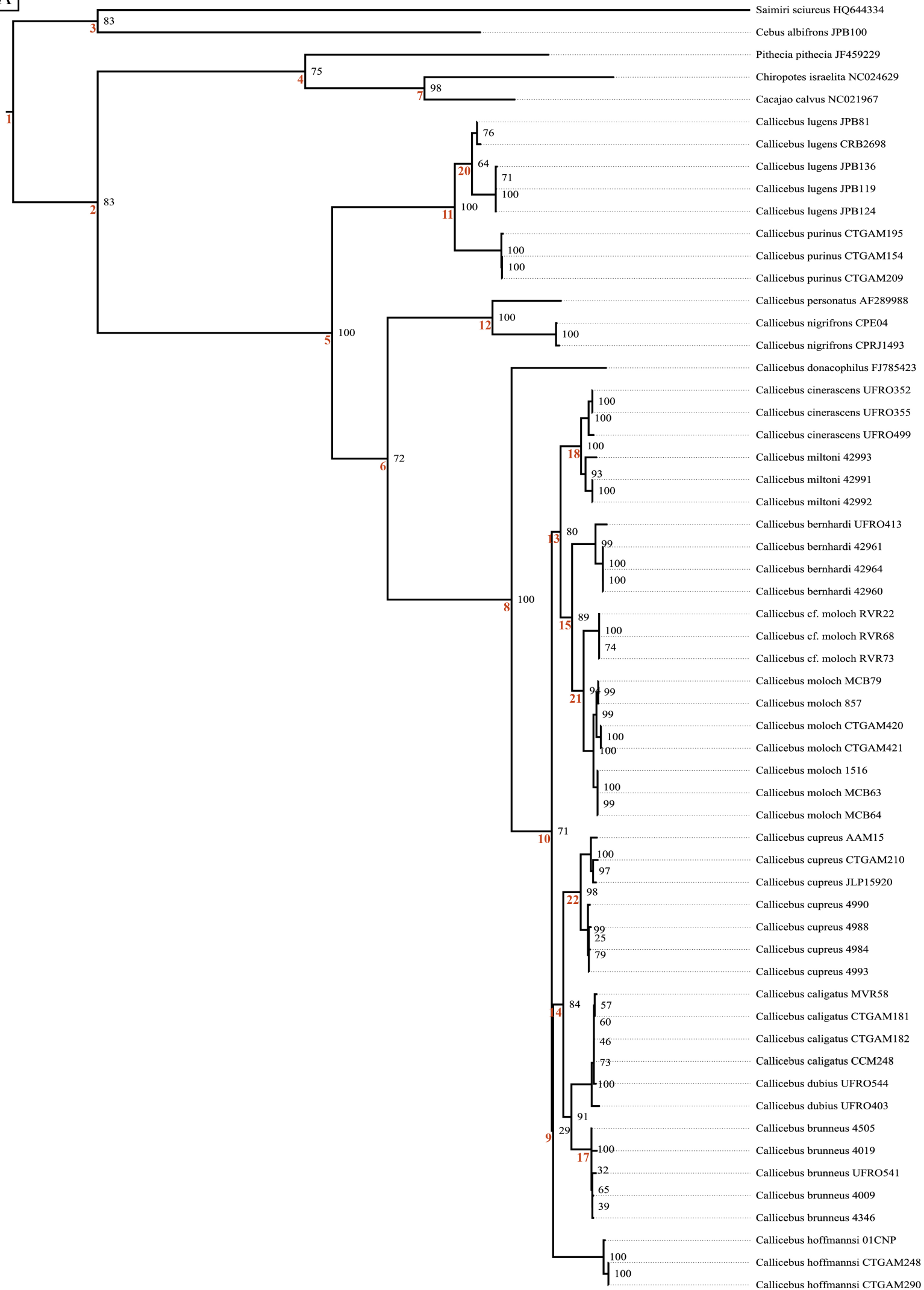

B

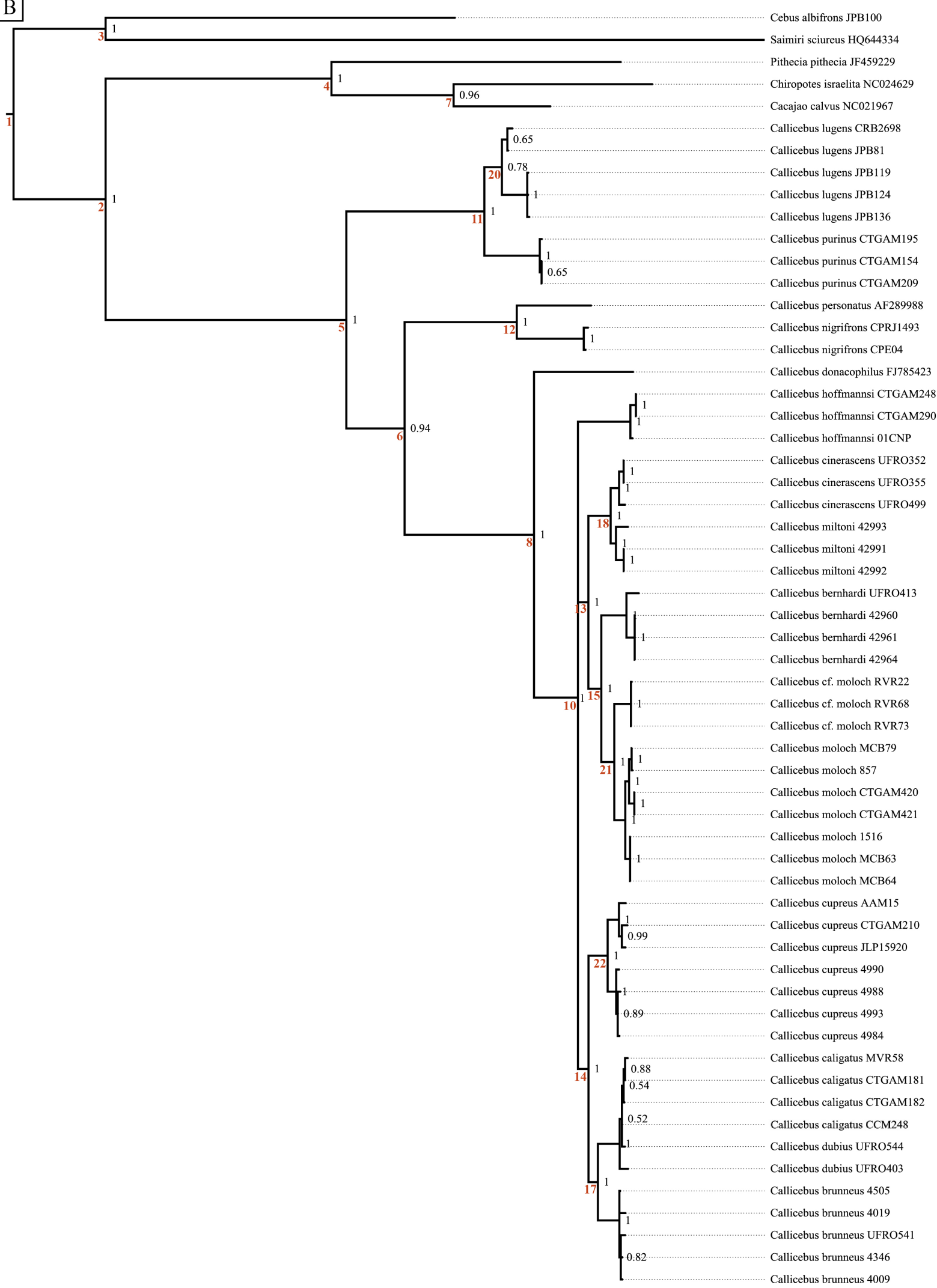

0.05

Supplement: Additional file 4: — Phylogenetic trees inferred from the mitochondrial dataset. Shown are the phylogenetic trees with node support values based on maximum likelihood (A: RAxML) and Bayesian (B: MrBayes) methods of analysis. Red numbers represent nodes of interest listed in Additional file 1. (PDF 2750 kb) [file 12983_2016_142_MOESM4_ESM.pdf]

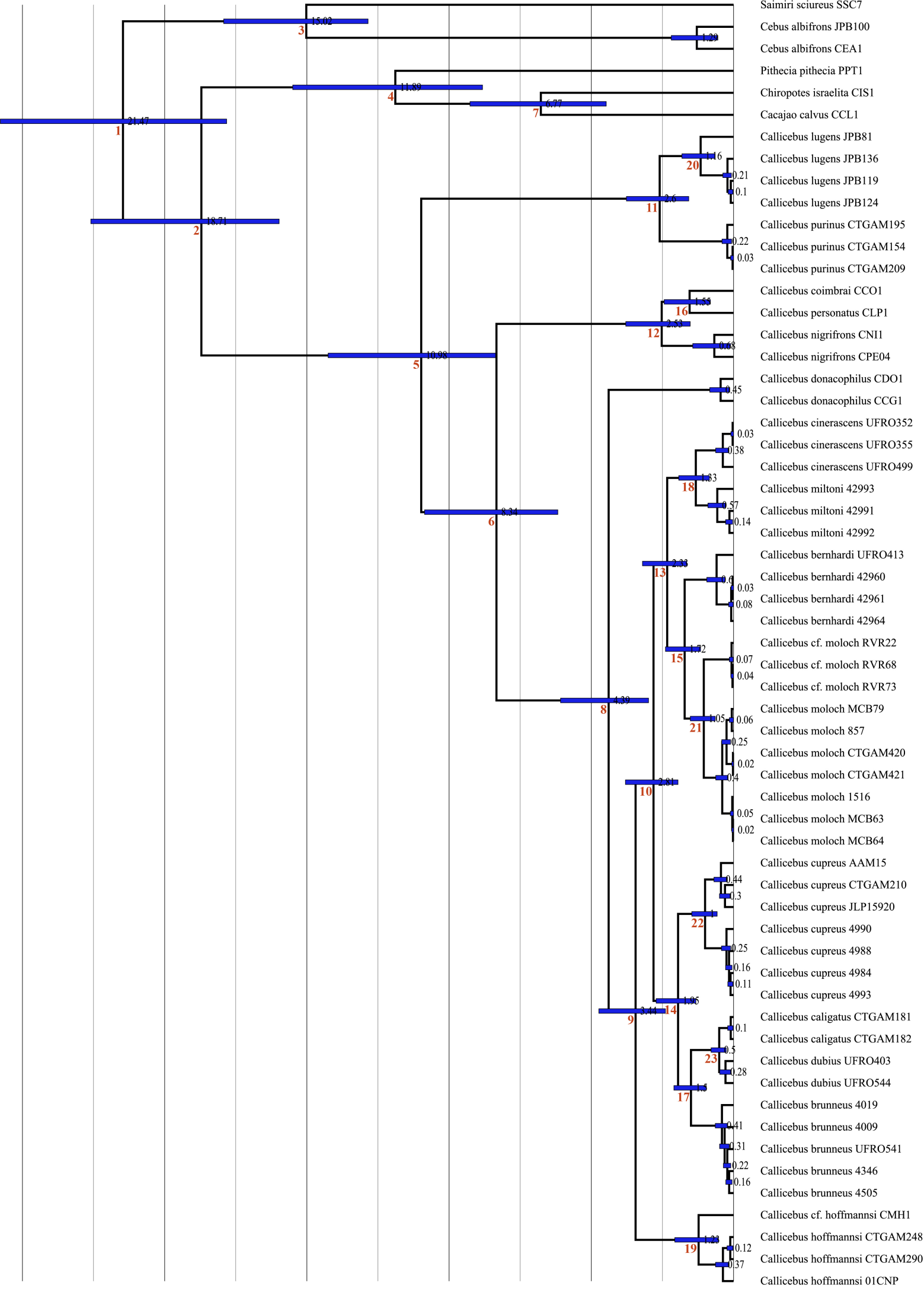

Supplement: Additional file 6: — BEAST time-calibrated phylogeny inferred from the combined dataset. Node bars indicate the 95 % highest posterior density. Red numbers represent nodes of interest listed in Additional file 1. (PDF 1903 kb) [file 12983_2016_142_MOESM6_ESM.pdf]

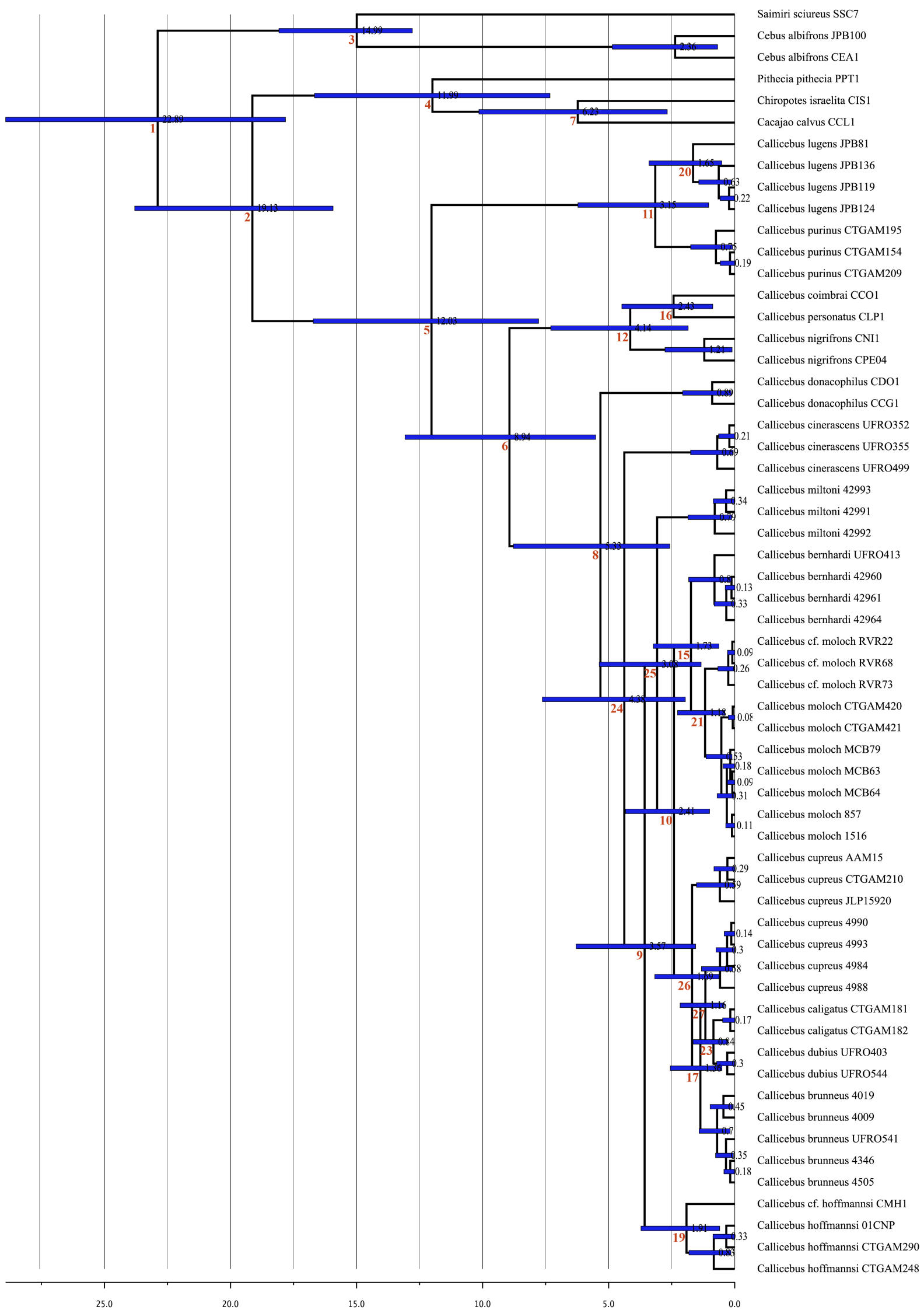

Supplement: Additional file 7: — BEAST time-calibrated phylogeny inferred from the nuclear dataset. Node bars indicate the 95 % highest posterior density. Red numbers represent nodes of interest listed in Additional file 1. (PDF 1952 kb) [file 12983_2016_142_MOESM7_ESM.pdf]
